# Mackinawite nanozymes as reactive oxygen species scavengers for acute kidney injury alleviation

**DOI:** 10.1186/s12951-023-02034-7

**Published:** 2023-08-19

**Authors:** Zhuobin Xu, Yufei Zhu, Mengke Xie, Kankan Liu, Liangliang Cai, Huihui Wang, Dandan Li, Hao Chen, Lizeng Gao

**Affiliations:** 1 Institute of Translational Medicine, Medical College, Yangzhou University, Yangzhou, 225009 China; 2 Jiangsu Key Laboratory of Integrated Traditional Chinese and Western Medicine for Prevention and Treatment of Senile Diseases, Yangzhou University, Yangzhou, 225009 China; 3 Department of Orthopedics, Affiliated Hospital of Yangzhou University, Yangzhou, 225009 Jiangsu China; 4 CAS Engineering Laboratory for Nanozyme, Institute of Biophysics, Chinese Academy of Sciences, Beijing, 100101 China

**Keywords:** Mackinawite nanozymes, Iron sulfides, Reactive oxygen species, Acute kidney injury

## Abstract

**Background:**

Iron sulfide nanomaterials have been successfully employed as therapeutic agents for bacterial infection therapy and catalytic-ferroptosis synergistic tumor therapy due to their unique structures, physiochemical properties, and biocompatibility. However, biomedical research and understanding of the biological functions of iron sulfides are insufficient, and how iron sulfide nanomaterials affect reactive oxygen species (ROS) in diseases remains unknown. Acute kidney injury (AKI) is associated with high levels of ROS, and therefore nanomedicine-mediated antioxidant therapy has emerged as a novel strategy for its alleviation.

**Results:**

Here, mackinawite nanozymes were synthesized from glutathione (GSH) and iron ions (Fe^3+^) (denoted as GFeSNs) using a hydrothermal method, and then evaluated as ROS scavengers for ROS-related AKI treatment. GFeSNs showed broad-spectrum ROS scavenging ability through synergistic interactions of multiple enzymes-like and hydrogen polysulfide-releasing properties. Furthermore, both in vitro and in vivo experiments demonstrated that GFeSNs exhibited outstanding cytoprotective effects against ROS-induced damage at extremely low doses and significantly improved treatment outcomes in AKI.

**Conclusions:**

Given the synergetic antioxidant properties and high biocompatibility, GFeSNs exhibit great potential for the treatment of AKI and other ROS-associated diseases.

**Graphical Abstract:**

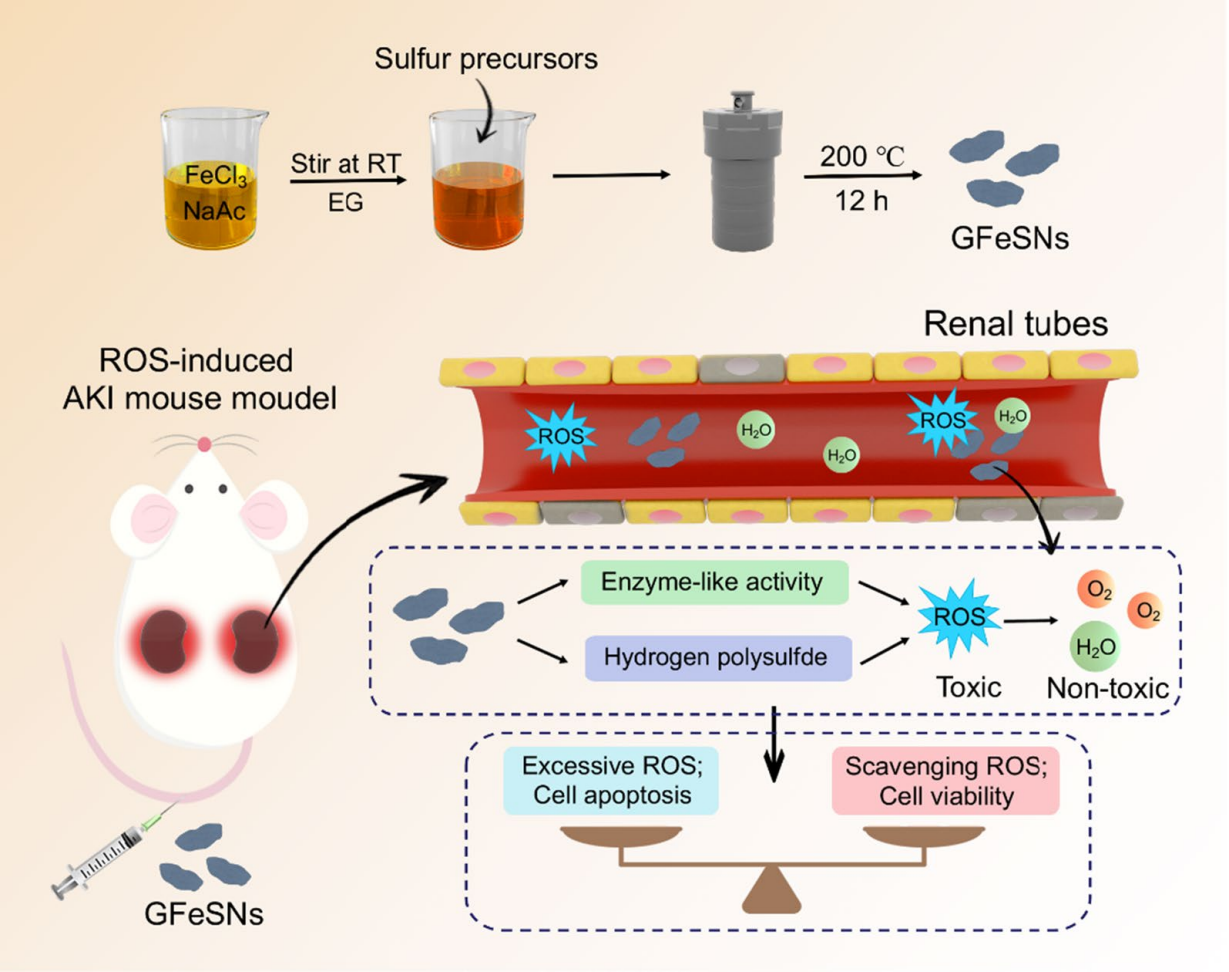

**Supplementary Information:**

The online version contains supplementary material available at 10.1186/s12951-023-02034-7.

## Background

Acute kidney injury (AKI) is a prominent clinical disease characterized by a rapid decrease in renal excretory function accompanied by decreased urine output and increased nitrogen metabolism accumulation [1, 2]. AKI can be caused by a variety of injuries, including ischemia–reperfusion, sepsis [3, 4], hypotension [5, 6], and overuse of antibiotics and chemotherapeutics [7], and is a growing healthcare concern worldwide [8]. AKI is one of the most common clinical complications, occurring in nearly 46% of hospitalized patients with COVID-19 [9, 10], a higher rate than in non-COVID-19 patients. AKI patients with COVID-19 also face a reduced likelihood of renal function recovery and increased risk of death [2, 11, 12]. However, apart from hemodialysis and kidney transplantation, there is currently a lack of effective therapies to prevent the development of AKI or promote its reversal [13, 14].

Pathological analysis of AKI suggests that reactive oxygen species (ROS) can trigger oxidative stress and inflammation, causing damage to lipids, nucleic acids, and proteins, which are closely related to the occurrence of AKI [15, 16]. While low levels of ROS generated during renal metabolism in healthy kidneys are beneficial for physiological redox signaling to promote cell survival [17, 18], proliferation, and growth, excessive ROS can cause oxidative stress damage in cells [19], further stimulating the activation of interstitial fibroblasts, leading to renal fibrosis, chronic kidney disease [20], and eventually irreversible and permanent damage to renal function [21]. Therefore, effective ROS clearance may be the key to AKI prevention and treatment [22, 23].

Although small-molecule antioxidants such as N-acetyl cysteine (NAC) and amifostine (AMF) show positive effects in preventing AKI, poor efficacy and potential toxicity risks limit their clinical application [16, 24, 25]. Recent advances in nanotechnology have developed a class of nanomaterials with ROS scavenging ability for AKI antioxidant therapy [26, 27], including molybdenum-based nanoclusters [28], DNA nanodevices [29, 30], carbon quantum dots [31, 32], melanin nanoparticles [33, 34], black phosphorus nanosheets [35], polyphenol-based nanoparticles [36], ultrasmall copper-based nanoparticles [24], and Prussian blue nanoparticles [37]. Iron-based nanomaterials have been widely employed in biomedicine due to their biocompatibility and biological function [38–40]. For instance, iron oxide nanoparticles (ferumoxytol) have been approved by the Food and Drug Administration (FDA) as iron supplement for treating iron deficiency, and as contrast agents for magnetic resonance imaging [41–43]. Furthermore, iron oxide and iron sulfide nanoparticles have been widely used in antibacterial and antitumor therapy via intrinsic enzyme-like activity, accompanied by ferroptosis-like properties [44, 45]. We recently found that iron sulfide nanomaterials (FeSNs) can continuously and steadily release hydrogen polysulfides, with strong anti-inflammatory and antioxidant properties [46]. Polysulfides rapidly scavenge the biological oxidant hydrogen peroxide (H_2_O_2_) and inhibit mitochondrial ROS production by modulating mitochondrial redox signaling [47]. The hydrogen polysulfide donor Na_2_S_4_ is also a promising therapeutic agent for the treatment of cisplatin-induced nephrotoxicity and as an adjuvant to cisplatin for cancer therapy [48].

Here, we screened several active sulfides, including L-cysteine, diallyl trisulfide (DATS), glutathione (GSH), and sodium thiosulfate, to hydrothermally synthesize into iron sulfides (CFeSNs, DFeSNs, GFeSNs, and TFeSNs) with lowly toxic ferric ions. The as-synthesized GFeSNs showed the strongest ability to suppress superoxide anions and release hydrogen polysulfides and were therefore used for further study. Given their synergistic mode of action, GFeSNs exhibited broad-spectrum ROS scavenging activities, including against hydroxyl radical (**·**OH), superoxide anion (O_2_^•−^), and hydrogen peroxide (H_2_O_2_), and protected cells from oxidative damage in vitro. GFeSNs showed high biocompatibility and strong ROS scavenging ability at a working concentration of 5 μg/mL in vitro and 500 μg/kg for AKI in vivo. To the best of our knowledge, this dose is much lower than that of any other iron sulfide compounds reported for the treatment of bacterial infections or tumors [49–51]. This study provides a new approach for preparing nano-sized mackinawite nanozymes with robust ROS scavenging capability. These as-prepared novel mackinawite nanozymes show considerable application potential as efficient antioxidant agents to meet the clinical needs of AKI and other ROS-related therapy.

## Materials and methods

### Materials

Ferric chloride (FeCl_3_), GSH, L-cysteine, DATS, sodium thiosulfate, 3,3′,5,5′-tetramethylbenzidine (TMB), H_2_O_2_, and NAC were purchased from Aladdin Biochemical Technology (Shanghai, China). 2′,7′-Dichlorofluorescein diacetate (DCFH-DA) and sodium acetate trihydrate (NaAc·3H_2_O) were purchased from Sigma Aldrich (Shanghai, China). Ethylene glycol (EG) and 5,5-dimethyl-1-pyrrooline N-oxide (DMPO) were obtained from Macklin (Shanghai, China). SulfoBiotics-SSP4 was purchased from DOJINDO (Japan). Phosphate-buffered saline (PBS, pH 7.4) solution was prepared in the laboratory. All chemicals and reagents were of analytical grade. Fetal bovine serum (FBS) and Dulbecco’s Modified Eagle Medium (DMEM) were purchased from HyClone (Logan, USA).

### Synthesis of GFeSNs

FeCl_3_ (5 mmol) was dissolved in EG (40 mL) with magnetic stirring for 15 min, after which NaAc·3H_2_O (40 mmol) and GSH (4 mmol) were slowly added with continuous stirring until the solution became transparent. The solution was then transferred to a polytetrafluoroethylene reactor and reacted at 200 ℃ for 12 h. The reactant was then washed with ethanol absolute and ultrapure water three times each. Finally, the product was freeze-dried overnight and stored at 4 ℃ until further use.

### H_2_O_2_ scavenging activity of GFeSNs

The H_2_O_2_ scavenging effect of GFeSNs at different concentrations (2.5–80 μg/mL) was examined using a hydrogen peroxide detection kit (Nanjing Jiancheng Bioengineering Institute, China). In brief, GFeSNs were incubated at a range of concentrations (2.5–80 μg/mL) with 500 mM H_2_O_2_ for 24 h at 37 °C, respectively, after which the samples were reacted with ammonium molybdate and measured at 405 nm.

### ·OH scavenging activity of GFeSNs

The **·**OH generated by the Fenton reaction reacts with salicylic acid to generate 2,3-dihydroxybenzoic acid with special absorption at 510 nm, with the reaction formula: H_2_O_2_ + Fe^2+^  = **·**OH + H_2_O + Fe^3+^. Here, 9 mM salicylic acid and different concentrations of GFeSNs (2.5–80 μg/mL) were successively mixed, followed by the addition of an appropriate amount of deionized water and 8.8 mM H_2_O_2_. Absorbance was measured at 510 nm after incubation in a water bath at 37 °C for 15 min.

Electron paramagnetic resonance (EPR) spectral signals were measured using a Bruker A300 spectrometer (Bruker, Germany). Typically, 1 mM H_2_O_2_, 20 μM FeSO_4_, 100 mM DMPO, and different concentrations of GFeSNs (0, 10, and 20 μg/mL) were added to acetic acid-sodium acetate (HAc-NaAc) buffer (0.1 M, pH 7), with the EPR signal then immediately detected.

### O_2_^•−^ scavenging activity of GFeSNs

The GFeSN scavenging activity of O_2_^•−^ was assessed using a superoxide anion assay kit (Beyotime Biotechnology, Shanghai, China) according to the manufacturer’s instructions. Different concentrations of GFeSNs (2.5–80 μg/mL) were added to the working solution. After standing for 30 min, absorbance was measured at 560 nm using a multiple plate reader. EPR spectroscopy further confirmed the superoxide dismutase (SOD)-like activity of GFeSNs. Briefly, superoxide anion radicals were produced by illuminating riboflavin solution, with 2 mM riboflavin, 100 mM DMPO, and different concentrations of GFeSNs (0, 10, and 20 μg/mL) added to the buffer and EPR signals recorded immediately.

### ABTS radical scavenging activity of GFeSNs

The ABTS (2,2′-azinobis(3-ethylbenzthiazoline-6-sulfonate) radical scavenging capacity of GFeSNs was assessed using a superoxide anion assay kit (Beyotime Biotechnology, Shanghai, China). Different concentrations of GFeSNs (2.5–80 μg/mL) were added to the working solution, with the ABTS radical scavenging abilities then calculated according to the manufacturer’s instructions.

### DPPH radical scavenging activity of GFeSNs

Different concentrations of GFeSNs (0, 5, 10, 20, 40, 80, and 160 μg/mL) were mixed with 125 μM DPPH (1-diphenyl-2-picrylhydrazyl) in ethanol for 30 min at the same volume, after which absorbance was measured at 517 nm. The final GFeSN concentrations were 0, 2.5, 5, 10, 20, 40, and 80 μg/mL and the final DPPH concentration was 62.5 μM.

### Cell culture

Normal rat kidney-52E (NRK-52E) cells and human embryonic kidney 293 (HEK293) cells were purchased from the American Type Culture Collection (ATCC). The cells were cultured in DMEM (10% FBS and 1% penicillin/streptomycin) at 37 °C in an incubator under a humidified atmosphere of 5% CO_2_.

### Cell viability assay

GFeSN cytotoxicity was determined by the cell counting kit-8 assay in vitro. The HEK293 and NRK-52E cells were seeded into 96-well cell culture plates at a density of 10^4^ cells/well, then incubated at 37 °C in an incubator with 5% CO_2_ overnight. After incubation with different concentrations of GFeSNs (0, 2.5, 5, 10, and 20 µg/mL) for 24 h, the cell culture medium was removed and 100 µL of CCK-8 solution was added to the plate for 2.5 h incubation at 37 °C. Absorbance was measured at 450 nm using a microplate reader and cell viability was then calculated.

### Mitochondrial membrane potential (MMP) assay

A loss of MMP assay was carried out using a JC-1 (5,5′,6,6′-Tetrachloro-1,1′,3,3′-tetraethylimidacarbocyanine) mitochondrial membrane potential assay kit (Beyotime Biotechnology, Shanghai, China). NRK-52E cells were seeded into a 6-well plate at a density of 2 × 10^5^ cells/well and cultured at 37 °C under 5% CO_2_ overnight. The cells were pretreated with PBS or GFeSNs (5 or 10 μg/mL) for 2 h, then incubated with H_2_O_2_ (300 μM) for a further 24 h at 37 °C under 5% CO_2_. Finally, the cells were washed with PBS before the addition of JC-1, followed by 20 min of incubation at 37 °C. After washing with the cell culture medium, the cells were imaged using a fluorescence microscope.

### In vitro ROS scavenging of GFeSNs

To investigate the ROS scavenging ability of GFeSNs, NRK-52E cells were seeded into 96- and 24-well plates at densities of 10^4^ cells/well and 10^5^ cells/well, respectively. After 24 h of incubation, different concentrations of GFeSNs (0–10 μg/mL) were added to each well and incubated for 30 min. Cells seeded in the 96-well plates were treated with 300 μM or 500 μM H_2_O_2_ and further incubated at 37 °C for 24 h. After that, the cell culture medium was removed and 100 µL of CCK-8 solution was added to the plate and incubated for 2.5 h at 37 °C. Absorbance was measured at 450 nm using a microplate reader. Wells without the addition of H_2_O_2_ were regarded as the negative control.

DCFH-DA was used to detect intracellular ROS levels. After pretreatment with different concentrations of GFeSNs (0–10 μg/mL) for 30 min, cells seeded in 6-well plates were further incubated with 300 μM H_2_O_2_ at 37 °C for 24 h. Cells were gently washed three times with serum-free medium, then incubated with 10 μM DCFH-DA in serum-free medium in the dark at 37 °C for 30 min. The cells were then washed thrice with serum-free medium and imaged using a fluorescence microscope.

### In vivo biocompatibility evaluation of GFeSNs

All animal procedures were performed in accordance with the Guidelines for Care and Use of Laboratory Animals of Yangzhou University and approved by the Animal Ethics Committee of Medical College of Yangzhou University. Female BALB/c mice (aged 4–6 weeks, obtained from Comparative Medicine Centre of Yangzhou University) were intravenously administered a single dose of 500 μg/kg GFeSNs. Mice injected with PBS were used as the control group. At 1 day post injection, blood samples were collected for complete blood panel analysis and serum biochemical tests. At 7th and 30th day post injection, the mice were sacrificed to harvest major organs (heart, liver, spleen, lung, and kidney) for hematoxylin and eosin (H&E) staining and histological analysis.

### In vivo therapeutic outcome of GFeSNs on AKI mice

Female BALB/c mice (6–8 weeks, 20–25 g) were deprived of water for 15 h. Subsequently, their two hindlimbs were intramuscularly injected with 50% glycerol equally at a dose of 8 mL/kg, followed by free access to water and food. The mice were randomly divided into five groups (n = 5): control group, GFeSNs without glycerol group (500 μg/kg), AKI group, GFeSNs group (500 μg/kg), and NAC (10 mg/kg) group, respectively. Drugs and GFeSNs were administered once. Body weight variations in each group were monitored after treatment. At 24 h post glycerol injection, mice were sacrificed to collect renal tissues and blood samples for detecting serum creatinine (CRE) and blood urea nitrogen (BUN) levels. Kidney tissues were harvested to detect heme oxygenase-1 (HO-1), kidney injury molecule-1 (KIM-1), malondialdehyde (MDA), superoxide dismutase (SOD), lactate dehydrogenase (LDH), glutathione (GSH), interleukin 6 (IL-6), interleukin-1 beta (IL-1β), and tumor necrosis factor-alpha (TNF-a) levels.

Partial renal tissues from all groups were harvested and fixed with paraformaldehyde, then sectioned and stained with H&E. Other kidney samples from all groups were frozen and subsequently sectioned into tissue slices. The obtained sections were stained by 4′,6-diamidino-2-phenylindole (DAPI), dihydroethidium (DHE), or terminal deoxynucleotidyl transferase mediated dUTP nick-end labeling (TUNEL).

### Transcriptome analysis

Total RNA was extracted from PBS-treated (n = 5) and GFeSN-treated (n = 5) BALB/c mice using TRIzol extraction. RNA purification, reverse transcription, RNA sequencing, and bioinformatics data collection were performed by Majorbio Bio-Pharm Biotechnology Co., Ltd. (Shanghai, China).

### Statistical analysis

All data are shown as mean ± standard deviation (SD). Statistical differences between two groups were analyzed using unpaired Student’s *t-*test with GraphPad Prism v7. *P*-values less than 0.05 were considered statistically significant: *p* < 0.05; **p < 0.01; ***p < 0.001; ****p < 0.0001.

## Results and discussion

### Synthesis and characterization

**Fig. 1:**
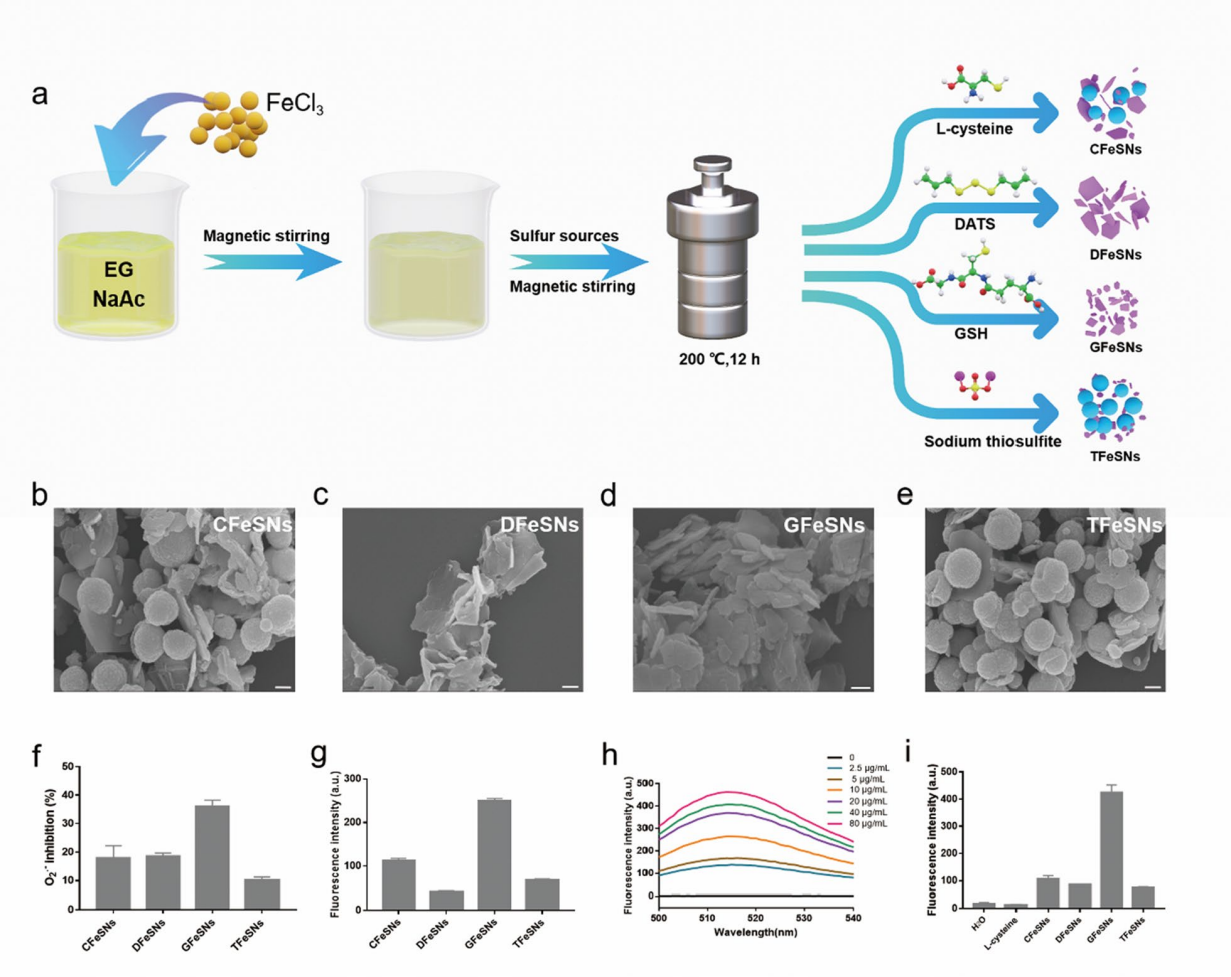
Preparation of iron sulfide nanoparticles. **a** Schematic illustration of different iron sulfide nanoparticles. SEM images of CFeSNs (**b**), DFeSNs (**c**), GFeSNs (**d**), and TFeSNs (**e**). Scale bar = 200 nm. **f** O_2_^−^ scavenging ability of four iron sulfide nanoparticles (10 μg/mL). **g** Identification of hydrogen polysulfides released from four iron sulfide nanoparticles (10 μg/mL) by SSP4 fluorescence probe. **h** The fluorescence spectra of GFeSNs after incubation with SSP4 for 30 min. **i** Relative fluorescence responses of 10 μg/mL iron sulfide nanoparticles and 50 μM WSP-5 incubated with or without 4 mM L-cysteine

GFeSNs, CFeSNs, DFeSNs, and TFeSNs were synthesized using the solvothermal method detailed in our previous study (Fig. 1a) [52]. As shown in the scanning electron microscopy (SEM) images (Fig. 1b–e), CFeSNs and TFeSNs displayed a mixture of spheres and sheets, while GFeSNs and DFeSNs exhibited free-standing nanosheets with a diameter of several hundred nanometers. It is found that the products with different morphologies are closely related to the different sulfur precursors introduced in the synthesis. In the previous literatures of our research group, there have been preliminary reports on the synthesis of iron sulfide nanoparticles with L-Cysteine and DATS as sulfur precursor [52, 53]. With the increase of sulfur precursor input, the proportion of spherical nanoparticles in the product decreased. Meanwhile only certain amount of GSH can be used as sulfur precursor to produce pure mackinawite FeS. Therefore, we concluded that the different sulfur sources are closely related to the composition and morphology of the iron sulfide products. According to the results, we speculate that sulfur sources with sulfhydryl and carbonyl group are the key raw material for the synthesis of pure mackinawite FeS by hydrothermal method. First, we evaluated the O_2_^•−^ clearance efficiency of the four iron sulfide nanoparticles. As shown in Fig. 1f, GFeSNs exhibited the strongest O_2_^•−^ scavenging ability compared with the other iron sulfide nanoparticles. Accumulating evidence suggests that hydrogen polysulfide-generating prodrugs can increase intracellular hydrogen polysulfide levels to regulate redox balance in cells, including protection of cells against harmful ROS levels and enhancing host antioxidant capacity [54, 55]. The hydrogen polysulfide-donating abilities of the four iron sulfide nanoparticles were detected using sulfane sulfur probe 4 (SSP4, 3′,6′-Di(O-thiosalicyl) fluorescein) at a concentration of 10 μg/mL. GFeSNs exhibited the strongest hydrogen polysulfide-producing ability among the four nanoparticles (Fig. 1g).

After incubation with SSP4 for 30 min, the fluorescence spectrum intensity of GFeSNs in PBS buffer were gradually enhanced with the increase of concentration (Fig. 1h). We also assessed hydrogen polysulfide release based on the ability of hydrogen polysulfides to rapidly react with L-cysteine to generate H_2_S.

GFeSNs reacting with L-cysteine showed the strongest fluorescence intensity among the iron sulfide nanoparticles. Incubation with either 10 mM phosphate buffer (pH 7.4) or L-cysteine alone generated limited fluorescence intensity (Fig. 1i). The release trend of hydrogen polysulfide from GFeSNs was then monitored. As shown in Additional file 1: Fig. S1**,** hydrogen polysulfide was released steadily and continuously over time in 12 h. To better understand the properties of GFeSNs, we monitored the release trend of iron ions in PBS by using the Iron Content Assay Kit. We found that the concentration of released iron ions in PBS rose rapidly in the first 2 h, and then continued to rise slowly until reaching the maximum concentration in 12 h (Additional file 1: Fig. S2), which was consistent with the release trend of hydrogen polysulfide.

**Fig. 2:**
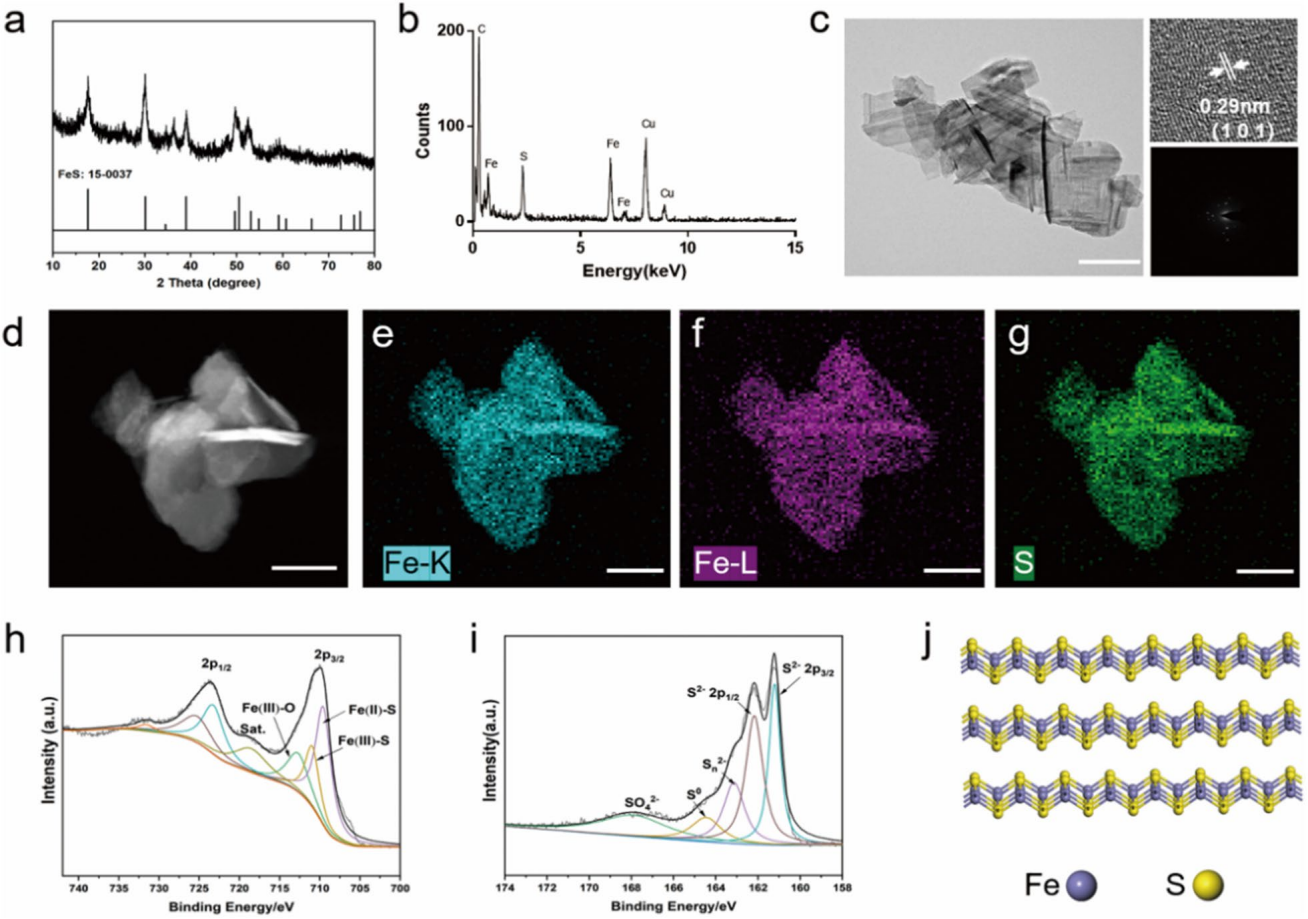
Characterization of GFeSNs. **a** XRD pattern of GFeSNs. **b** EDS analysis of GFeSNs. **c** High-resolution transmission electron microscope (HRTEM) image and selected-area electron diffraction (SAED) pattern of GFeSNs. Scale bar = 200 nm. **d** HAADF-STEM image of GFeSNs. Fe-K (**e**), Fe–L (**f**), and S (**g**) elemental mapping images of GFeSNs. Scale bar = 100 μm. Fe 2p (**h**) and S 2p (**i**) regions of XPS spectra of GFeSNs. **j** A optimized schematic atom structure represents crystal phases of GFeSNs

X-ray diffraction (XRD) pattern confirmed that GFeSNs were in pure phases of mackinawite FeS (PDF no. 15-0037) (Fig. 2a). The height of GFeSNs obtained from atomic force microscopy (AFM) measurement was approximately 55–62 nm (Additional file 1: Fig. S3). Elemental analysis by energy dispersive X-ray spectroscopy (EDS) revealed that the GFeSNs consisted of iron (Fe) and sulfur (S) (Fig. 2b). The high-resolution transmission electron microscope (HRTEM) image and selected area electron diffraction (SAED) analysis indicated that GFeSNs were flake-shaped with well-defined crystallinity, which exhibited clear lattice fringes with a d-spacing of 0.29 nm, corresponding to (101) crystal planes of mackinawite FeS (Fig. 2c). The STEM-energy dispersive X-ray spectroscopy (STEM-EDX) elemental mapping images of Fe-K, Fe–L, and S (Fig. 2d–g) clearly revealed the homogeneous distribution of Fe and S elements in GFeSNs. Furthermore, X-ray photoelectron spectroscopy (XPS) was used to analyze the surface chemical composition of the nanozymes (Fig. 2h, i) [56]. In the high-resolution spectra of Fe 2p_3/2_, the peak located at 709.6 eV corresponded to Fe(II)-S, and the peak at 711 eV was attributed to Fe(III)-S bonds, which was always observed at the surface of mackinawite. The peak at 712.7 eV was assigned to Fe(III)-O species, suggesting surface oxidation of the nanozymes in air. With respect to the S 2p spectra, the peaks at 161.2 eV and 162.1 eV were related to S 2p_3/2_ and S 2p_1/2_ of S^2−^, while the peaks at 163.1 eV, 164.4 eV, and 167.9 eV were assigned to oxidized sulfur species of S_n_^2−^, S^0^, and SO_4_^2−^, respectively. The optimized bulk structure of GFeSNs were computer-simulated and displayed in Fig. 2j.

### ROS scavenging activities of GFeSNs

**Fig. 3:**
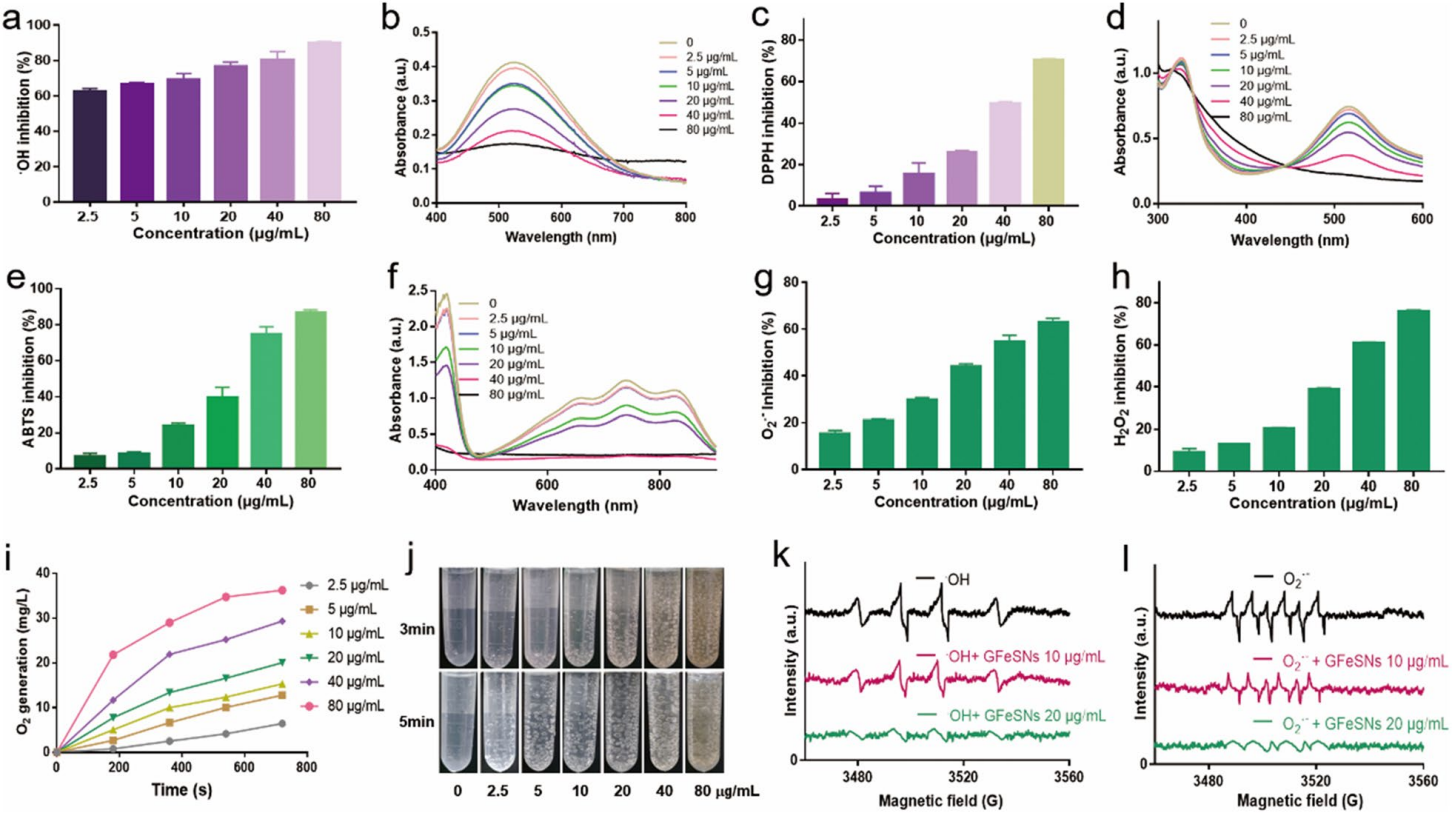
ROS scavenging ability of GFeSNs. GFeSN scavenging ability against **·**OH (**a**), DPPH (**c**), ABTS (**e**), O_2_^−^ (**g**), and H_2_O_2_ (**h**). The ultraviolet–visible (UV–vis) spectra of free radicals **·**OH (**b**), DPPH (**d**), and ABTS (**f**) incubated with GFeSNs at different concentrations (0–80 μg/mL). **i** O_2_ generation from H_2_O_2_ catalyzed by GFeSNs. **j** Images of GFeSNs (0–80 μg/mL) catalyzing formation of O_2_ bubbles. ESR spectra of GFeSNs reacted with **·**OH (**k**) and O_2_^•−^ (**l**)

Given the major role of ROS in AKI prevention and treatment, representative ROS, such as ·OH, O_2_^•−^, and H_2_O_2_, were used to systematically evaluate the antioxidant abilities of GFeSNs [57]. As seen in Fig. 3, GFeSNs showed high concentration-dependent ROS scavenging activity. GFeSNs significantly removed ·OH by inhibiting salicylic acid oxidation (Fig. 3a, b). Notably, 60% of ·OH was scavenged at a GFeSN concentration of 2.5 μg/mL. Methylene blue (MB) was used to detect the ·OH radicals generated by the Fenton reaction. MB was degraded to a colorless state in the presence of ·OH. However, MB remained blue under the protection of GFeSNs, indicating that the nanoparticles possessed strong ·OH radical scavenging ability (Additional file 1: Fig. S4). The antioxidant capacity of GFeSNs against DPPH and ABTS radicals was evaluated. As shown in Fig. 3c, d, the absorbance of DPPH at 517 nm markedly decreased after mixing with various concentrations of GFeSNs (2.5, 5, 20, 40, and 80 μg/mL). Similarly, as shown in Fig. 3e, f, > 70% of ABTS was scavenged by GFeSNs at a concentration of 40 μg/mL. Thus, GFeSNs demonstrated strong radical scavenging properties. GFeSNs further showed strong antioxidant activity against O_2_^•−^ and H_2_O_2_, scavenging more than 62% of O_2_^•−^ (Fig. 3g) and 76% of H_2_O_2_ (Fig. 3h) at a concentration of 80 µg/mL. In comparison, GSH exhibited negligible antioxidant activity in vitro (Additional file 1: Fig. S5). We also measured the O_2_^•−^ radical scavenging ability of GFeSNs in aqueous solution after 24 and 48 h, with results showing that GFeSNs still removed more than 50% of O_2_^•−^ (Additional file 1: Fig. S6). Given its longer half-life than O_2_^•−^ and ·OH, H_2_O_2_ is considered one of the most important ROS and exhibits the highest intracellular concentration. As such, we next investigated the concentration-dependent catalase (CAT)-like activity of GFeSNs (Additional file 1: Fig. S7), which was further assessed by monitoring dissolved O_2_. A gradual increase in O_2_ concentration was observed in the presence of GFeSNs (Fig. 3i). Consistent with this, the formation of many O_2_ bubbles were photographed after the addition of GFeSNs to 1000 mM H_2_O_2_ solution in 0.1 M NaAc (pH = 7) at 37 ℃ (Fig. 3j). We further investigated the multiple enzyme-mimicking properties of GFeSNs under different pH conditions*.* GFeSNs exerted SOD-like and CAT-like activity in pH-independent manners. The results revealed that GFeSNs possess SOD-like activity under both neutral and acidic conditions but with much better performance at neutral conditions. Similarly, under acidic conditions (pH = 6.5/5.5), the decomposition efficiency of H_2_O_2_ catalyzed by GFeSNs was significantly reduced. Intriguingly, the POD-like activity of GFeSNs was detected under different pH conditions. The results revealed that GFeSNs possess weak POD activity, which could hardly convert H_2_O_2_ into toxic free radicals even in the acidic environment (Additional file 1: Fig. S8). Electron spin resonance (ESR) spectra were used to detect the ·OH generated by the Fenton reaction and O_2_^•−^ generated from riboflavin exposed to light, using DMPO as the trapping agent of free radicals. Notably, ESR signals of ·OH and O_2_^•−^ decreased significantly upon the addition of 10 and 20 μg/mL GFeSNs (Fig. 3k, l). At present, the hydrogen polysulfide donors reported in the literatures are mainly small molecular compounds, with complex synthesis steps, poor stability and rapid release of hydrogen polysulfide in solution [46, 58, 59]. GFeSNs is a new type of polysulfide donor, which is easy to be synthesized in large quantity, easy to be preserved and well tolerated. More strikingly, GFeSNs can release hydrogen polysulfide steadily and continuously over time during degradation and exert antioxidant enzyme activity. These results indicate that GFeSNs are strong ROS scavenging agents.

### GFeSN-mediated protection of H_2_O_2_-stimulated cells

**Fig. 4:**
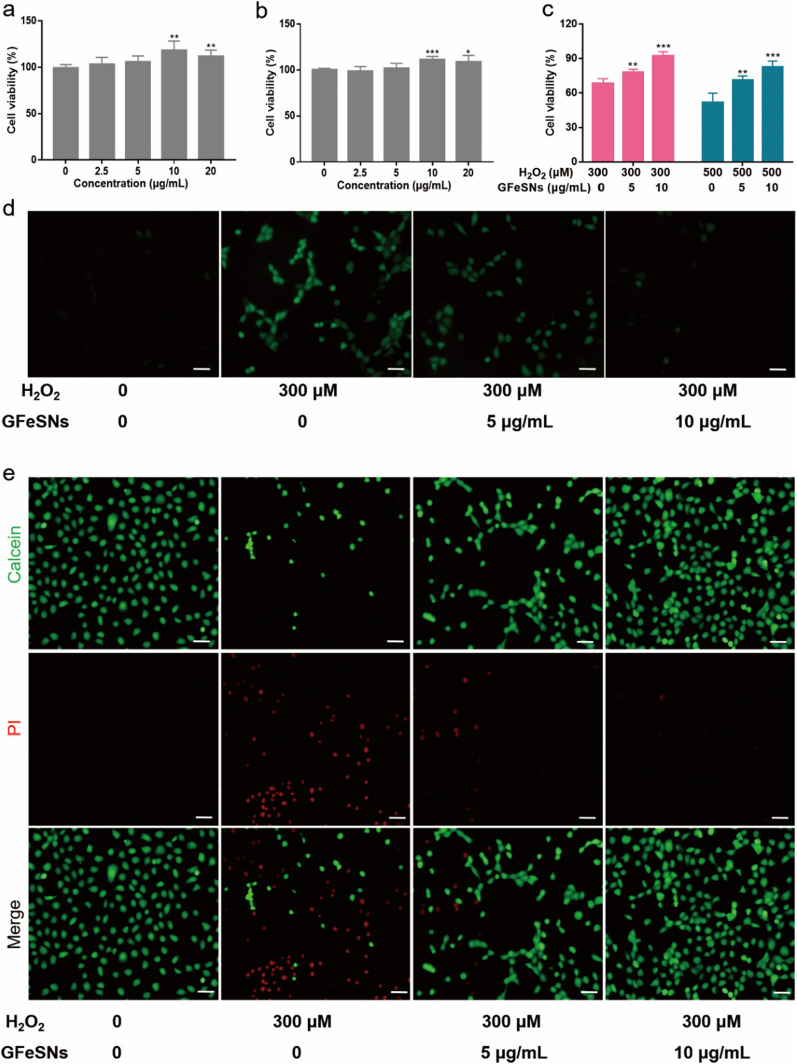
ROS scavenging ability of GFeSNs in vitro. In vitro NRK-52E (**a**) and HEK293 (**b**) cell viability under different treatment conditions (n = 5). **c** NRK-52E cell viability under H_2_O_2_ treatment (0.3 and 0.5 mM) with or without GFeSN (5 and 10 μg/mL) pretreatment (n = 3). **d** Representative ROS staining (green fluorescence) of NRK-52E cells under different treatment conditions. Scale bar = 25 μm. **e** Representative Calcein AM/PI staining of NRK-52E cells under different treatment conditions. Scale bar = 25 μm. Error bars are mean ± SD (*p < 0.05, **p < 0.01 and ***p < 0.001)

The HEK293 and NRK-52E cells were used to detect the cytoprotective effects of GFeSNs under ROS damage in vitro. First, the CCK-8 assay results showed that GFeSNs were nontoxic to both cell lines at concentrations ranging from 2.5 to 20 μg/mL over 24 h. Surprisingly, GFeSNs promoted cell proliferation at concentrations of 10 and 20 μg/mL, which may be because hydrogen polysulfides can promote cell proliferation (Fig. 4a, b). Subsequently, NRK-52E cells were used to evaluate the cytoprotective properties of GFeSNs against ROS damage in vitro. As shown in Fig. 4C, compared to those without pretreatment, the viability of NRK-52E cells pretreated with 5 and 10 μg/mL GFeSNs for 1 h after treatment with 300 or 500 μM H_2_O_2_ significantly increased. Under 10 μg/mL GFeSN pretreatment, approximately 92% and 82% of cells were alive in the presence of 300 and 500 μM H_2_O_2_, respectively. The antioxidant performance of GFeSNs was further studied using the DCFH-DA probe. As seen in Fig. 4d.

ROS levels in the H_2_O_2_ group showed the highest signals. In contrast, when cells were pretreated with GFeSNs, intracellular ROS levels were significantly suppressed, We then examined the effect of GFeSNs on H_2_O_2_-induced cell death/apoptosis using Calcein AM/PI dual staining. As shown in Fig. 4e, the stained cells with Calcein AM/PI were found an increase in red fluorescence intensity after H_2_O_2_ treatment, and the addition of GFeSNs significantly reduced the ratio of dead cells induced by H_2_O_2_ treatment, indicating the cytoprotective properties of GFeSNs against ROS at the cellular level.

### GFeSNs mediated ROS scavenging and anti-inflammation in vitro

**Fig. 5:**
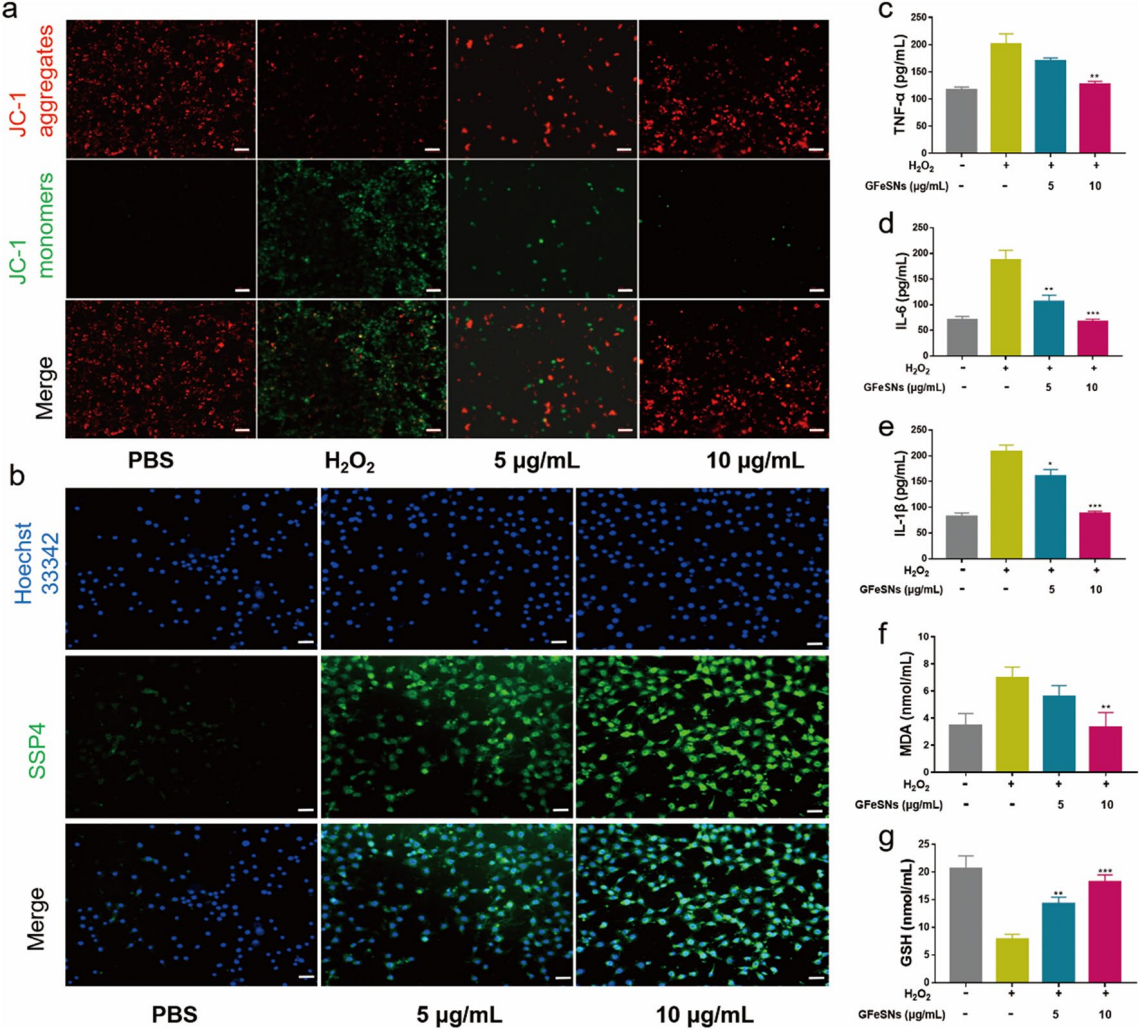
**a** Changes in MMP in NRK-52E cells after different treatment. Scale bar = 50 μm. **b** Representative SSP4 and Hoechst 33,342 staining of NRK-52E cells under different treatment conditions. Scale bar = 20 μm. TNF-α (**c**), IL-6 (**d**), IL-1β (**e**), MDA (**f**), and GSH (**g**) levels measured in NRK-52E cells under different treatment conditions (n = 3). Error bars are mean ± SD (*p < 0.05, **p < 0.01 and ***p < 0.001)

MMP is a key indicator of mitochondrial function. Excessive ROS can cause MMP loss, resulting in mitochondrial damage and fragmentation [25]. Here, changes in MMP were observed by JC-1 staining, determined by the transition between JC-1 aggregates (red fluorescence) and JC-1 monomers (green fluorescence) [60]. Results showed that green fluorescence signal intensity increased sharply in cells treated with H_2_O_2_, but decreased significantly in cells pretreated with GFeSNs, suggesting that GFeSNs can maintain mitochondrial function by inhibiting ROS-induced damage (Fig. 5a). We further characterized the entry of hydrogen polysulfide species into NRK-52E cells using the SSP4 probe. As shown in Fig. 5b, significant green fluorescence signals were observed in cells treated with GFeSNs compared to NRK-52E cells alone, indicating that GFeSNs releasing hydrogen polysulfide species entered the cells to alleviate intracellular oxidative stress. To better understand the ROS scavenging performance of GFeSNs at the cellular level, various biochemical measures of intracellular redox homeostasis were determined.

Several inflammatory cytokines involved in AKI progression were first examined, including TNF-α, IL-6 and IL-1β. As illustrated in Fig. 5c–e, H_2_O_2_ increased TNF-α, IL-6, and IL-1β levels in NRK-52E cells, while GFeSN treatment effectively decreased their expression. We then evaluated the level of lipid peroxidation and intracellular antioxidant status by assessing the level of intracellular MDA and GSH. Results showed that MDA levels increased and GSH levels decreased in the H_2_O_2_ group, while the PBS- and GFeSNs-treated groups showed no significant differences (Fig. 5f, g). These results strongly suggest that GFeSNs may exert synergistic anti-inflammatory and ROS scavenging effects to restore cellular redox balance.

### In vitro and in vivo biocompatibility of GFeSNs

SEM images were taken of GFeSNs placed in PBS solution for different times, and we found that GFeSNs degraded to small-sized lamellae after 24 h, indicating good degradability (Additional file 1: Fig. S9). As good red blood cell compatibility indicates high safety of intravenous administration [61], we studied hemolysis performance of GFeSN. Results showed that even at a high concentration, the hemolysis rate of GFeSNs was less than 5%, indicating high biocompatibility (Additional file 1: Fig. S10). Thus, GFeSNs were administered intravenously at a high dose of 1 mg/kg and in vivo toxicity was studied. H&E staining showed no obvious changes in the heart, liver, spleen, lung, and kidney at different time points (Additional file 1: Fig. S11). In addition, serum biochemical indicators including aspartate aminotransferase (AST), alanine aminotransferase (ALT) and blood routine indices in GFeSN-treated mice showed no obvious differences between PBS and GFeSN treated groups (Additional file 1: Fig. S12).

### In vivo therapeutic outcome of GFeSNs on AKI mice

Given the excellent anti-inflammatory and ROS scavenging abilities of GFeSNs, we speculated that the nanoparticles may be capable of protecting renal health from ROS damage, thereby alleviating AKI. As GFeSNs possessed a typical thin sheet-like morphology of hundreds of nanometers, the high planar/thickness ratio may facilitate accumulation in the kidney, thereby enabling efficient AKI therapy [62]. Furthermore, the hydrogen polysulfides generated by GFeSNs may be easily taken up by cells to play a synergistic role in maintaining cellular redox balance.

**Fig. 6:**
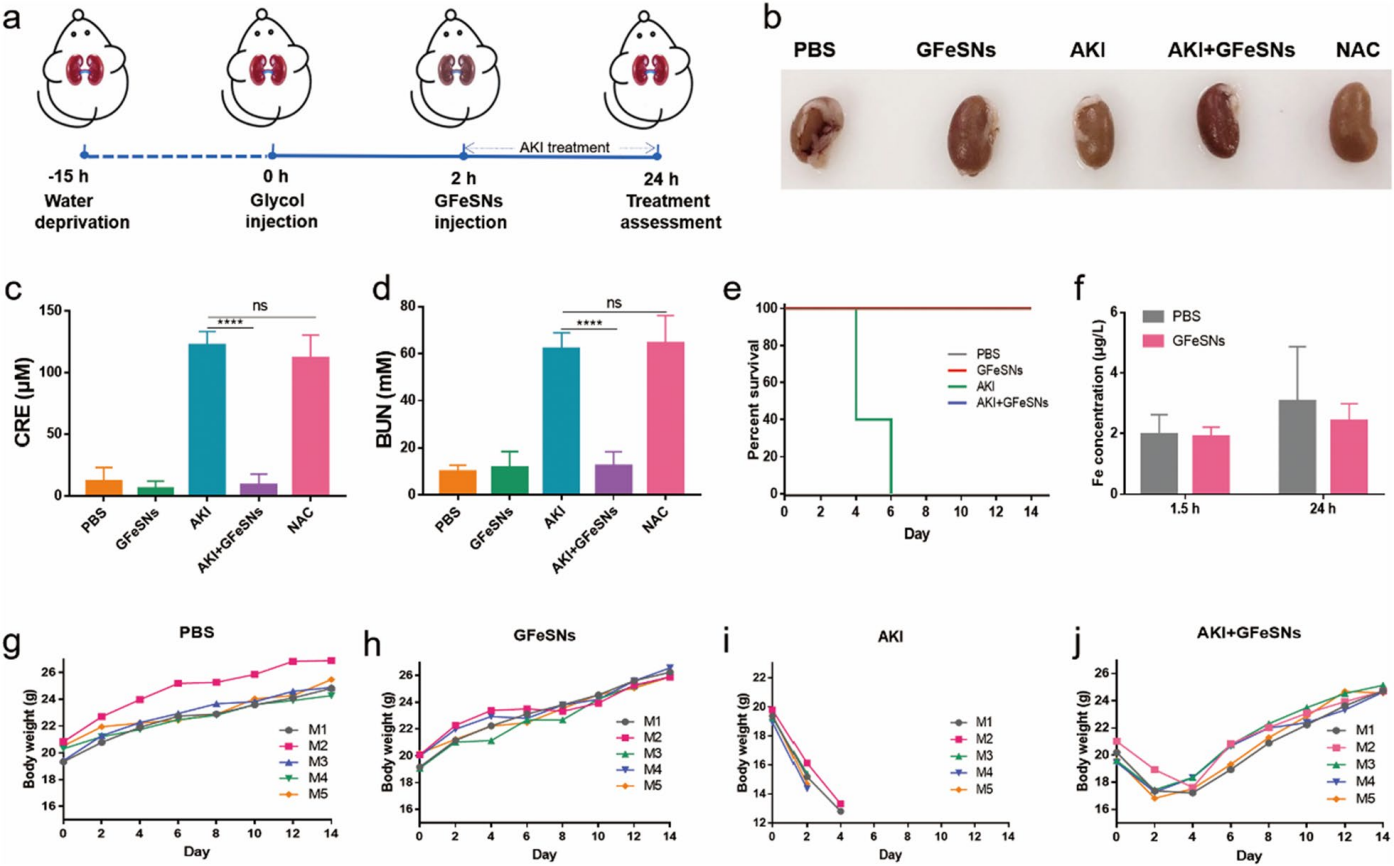
Evaluation of kidney function after GFeSN treatment. **a** Schematic for construction of AKI model mice and their treatment with GFeSNs. **b** Representative kidney samples from each group. **c** CRE levels in blood of mice after different treatments (n = 5). **d** BUN levels in mice after different treatments (n = 5). **e** Percentage of mouse survival under different treatments (n = 5). **f** Blood iron content in PBS- and GFeSN-treated mice at indicated time points post injection (n = 3). Body weight curves of PBS-treated healthy mice (**g**), healthy mice with intravenous injection of GFeSNs (**h**), AKI mice (**i**), and AKI mice with intravenous injection of GFeSNs (**j**) (n = 5). Error bars are mean ± SD (****p < 0.0001)

As illustrated in Fig. 6a, the AKI model was established by intramuscular injection of 50% glycerol into 15 h-dehydrated mice, which induced rhabdomyolysis and oxidative stress-related renal dysfunction. GFeSNs (500 μg/kg) were injected intravenously at 2 h post glycerol injection to verify their potential feasibility as antioxidants in the treatment of AKI. For comparison, NAC (20 mg/kg) was utilized as a positive control. The pictures of the corresponding kidneys were shown in Fig. 6b. The kidney of PBS-treated AKI mice showed a lighter color compared to PBS- and GFeSN-treated mice, while the kidney of GFeSN-treated AKI mice improved markedly. To further evaluate the therapeutic effect of GFeSNs, we measured the levels of two clinical renal excretion indicators (serum CRE and BUN) in different groups. AKI mice treated with PBS alone showed significantly high levels of CRE and BUN, while GFeSN-treated AKI mice showed significantly reduced levels of CRE and BUN. NAC treatment in AKI mice did not produce an obvious therapeutic effect (Fig. 6c, d). In addition, all AKI mice treated with PBS died within 6 days, whereas administration of GFeSNs significantly prolonged survival time beyond 14 days (Fig. 6e). Next, we evaluated the plasma concentration of GFeSNs after intravenous injection at 1.5 and 24 h by measuring the iron ion concentration. Results showed no significant difference between the PBS- and GFeSN-treated groups, indicating that GFeSNs exhibited therapeutic effects against AKI at very low concentrations (Fig. 6f). In addition, the AKI mice treated with GFeSNs showed sustained weight gain to normal levels after slight weight loss in the first few days, whereas AKI mice treated with PBS underwent sustained weight loss until death (Fig. 6g–j).

### Therapeutic mechanisms of GFeSNs on AKI

**Fig. 7:**
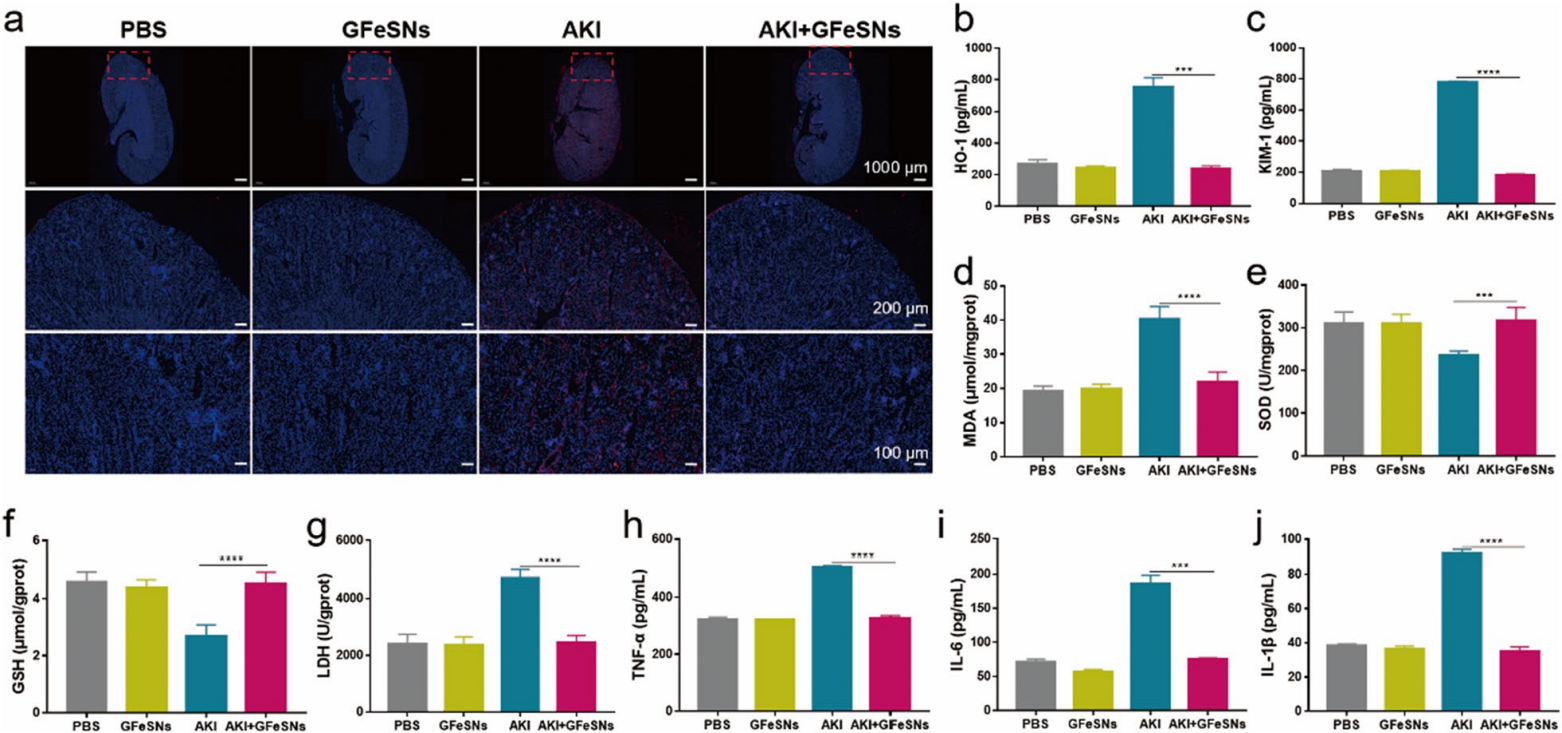
Therapeutic efficiency of GFeSNs on AKI. **a** DHE (red fluorescence) and DAPI (blue fluorescence) staining of kidney tissues from each group. HO-1 (**b**), KIM-1 (**c**), MDA (**d**), SOD (**e**), GSH (**f**), LDH (**g**), TNF-α (**h**), IL-6 (**i**), and IL-1β (**j**) levels measured in renal tissue homogenates from each group (n = 3). Error bars are mean ± SD (***p < 0.001 and ****p < 0.0001

Given the encouraging ROS scavenging capacity and biocompatibility of GFeSNs in vitro and in vivo, we investigated whether the ability of GFeSNs to treat AKI is correlated with ROS levels and antioxidant activities in renal tissues after treatment in AKI mice. We first stained kidney slices with DHE to detect superoxide generation. Compared with AKI mice, the superoxide levels in GFeSN-treated AKI mice were significantly reduced to normal mouse levels (Fig. 7a). In addition, we examined the renal expression levels of two important biomarkers of kidney injury, i.e., HO-1 and KIM-1. As shown in Fig. 7b, c, the levels of KIM-1 and HO-1 were significantly lower in GFeSN-treated AKI mice compared to AKI mice, approaching levels observed in normal mice. We then assessed antioxidant activity in renal tissues of AKI mice (Fig. 7d–f). MDA levels in the GFeSN-treated mice were consistent with those in healthy mice, whereas MDA levels were increased in the PBS-treated AKI mice. Furthermore, SOD and GSH levels in the GFeSN-treated AKI mice were restored to those similar in normal mice, while SOD and GSH levels in the AKI mice were significantly reduced. The protective effects of GFeSNs in AKI mice were also examined using LDH release assay (Fig. 7g). Our data showed that GFeSNs almost completely abolished LDH release in AKI mice to the same level as healthy mice, suggesting that GFeSNs can protect renal cells in AKI mice. To better understand the mechanism by which GFeSNs combat AKI, we evaluated the renal inflammatory response based on TNF-α, IL-6, and IL-1β levels using ELISA. As shown in Fig. 7h–j, GFeSN treatment reduced the release of proinflammatory factors TNF-α, IL-6, and IL-1β in AKI mice, indicating that GFeSNs can protect kidney tissues from oxidative stress by inhibiting the immune response during AKI.

**Fig. 8:**
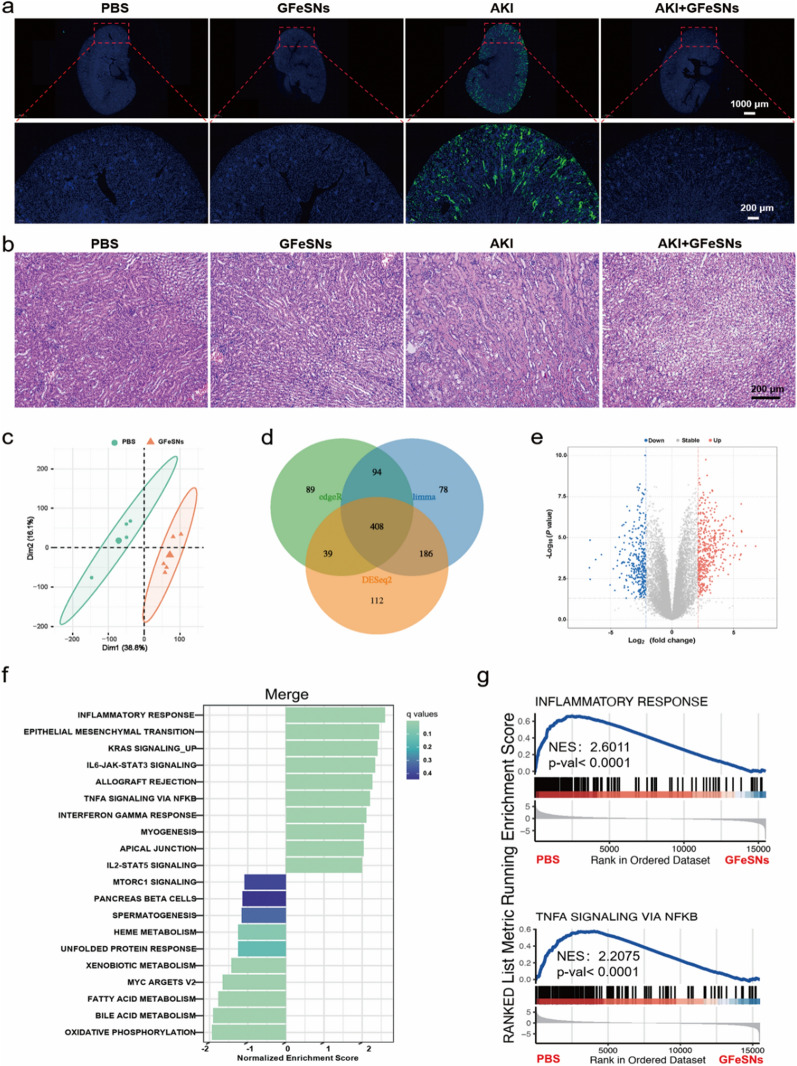
Therapeutic mechanisms of GFeSNs on AKI. **a** Fluorescence images of TUNEL-stained kidney sections from each group. **b** H&E staining of kidney tissues from each group. **c** PCA was performed on DEGs from the kidneys of GFeSN- and PBS-treated AKI mice (n = 5). Each data point corresponds to PCA of each sample. **d** Venn diagram of transcriptomic profiles of PBS- and GFeSN-treated AKI mice. **e** Volcano plots showing up-regulated and down-regulated genes by GFeSNs. **f** GSEA was performed to analyze enriched signaling pathways. Top 20 most significantly enriched pathways are shown. **g** GSEA identified two representative signaling pathways regulated by GFeSN treatment

Furthermore, the therapeutic effects of GFeSNs on AKI-induced apoptosis in the renal cortex were evaluated by TUNEL staining. As shown in Fig. 8a, massive apoptosis in the renal cortex of AKI mice was observed with loss of tubular structure. In contrast, no significant apoptosis was detected in the GFeSN-treated group, demonstrating the anti-apoptotic properties of GFeSNs against AKI, consistent with the renal function and blood analysis results. In addition, H&E staining of kidney tissues showed severe renal tubular injury with damaged tubules in AKI mice (Fig. 8b), whereas significantly reduced kidney damage was observed in the GFeSN-treated AKI mice. These results clearly demonstrate the outstanding therapeutic performance of GFeSNs against AKI and their potential as candidates for the treatment of ROS-related diseases.

To reveal the underlying therapeutic mechanisms of GFeSNs against AKI, we conducted RNA-seq profiling based on the GFeSN- and PBS-treated AKI mice. Principal component analysis (PCA) revealed substantially different transcriptomic profiles between the GFeSN- and PBS-treated AKI mouse kidneys (Fig. 8c). Given these differences, Deseq2, edgeR, and limma analyses were applied to identify significantly differentially expressed genes (DEGs), identifying 745, 630, and 766, respectively. The Venn diagram showed that a total of 408 DEGs were identified from the three methods (Fig. 8d). Volcano plots identified 766 significant DEGs, including 447 up-regulated and 319 down-regulated DEGs (Fig. 8e). Kyoto Encyclopedia of Genes and Genomes (KEGG) enrichment analysis and gene set enrichment analysis (GSEA) based on integrated differential expression and pathway analysis were performed to determine the effects of GFeSN-induced transcriptomic changes on biological functions and pathways (Fig. 8f, g). During the analysis, inflammation response exhibited significantly value. The significances were ranked by adjusted p-values using the limma algorithm and the top 10 enrichment (Fig. 8f) and 2 representative figures (Fig. 8g) were visualized. Consistent with the biochemical analyses, GFeSN-treated AKI mice kidneys exhibited the enrichment of inflammatory pathways including IL-6/JAK/STAT3 pathway, interferon (IFN)-γ and TNF-α signaling via NF-κB, demonstrating that the level of inflammation in the kidney of AKI mice was substantially decreased by GFeSN treatment, providing an improved environment for AKI alleviation.

## Conclusions

Here, we developed a novel mackinawite nanozyme synthesized from GSH and ferric ions using a hydrothermal method, which exhibited broad-spectrum ROS-eliminating activity against oxidative stress. In vitro experiments demonstrated that GFeSNs promoted cell proliferation and effectively protected cells from H_2_O_2_-induced oxidative stress. Our study suggested that the conversion of active sulfides into mackinawite nanozymes is an effective approach to enhance the ROS scavenging activity of sulfur-containing compounds. In addition, GFeSNs exhibited multiple enzyme-like activities and hydrogen polysulfide-releasing properties, which were strongly correlated with the capacity to scavenge superoxide anions. Therefore, the hydrothermal method may be an effective approach to convert sulfur and iron into metastable mackinawite nanozymes with high ROS scavenging ability. In addition, with high biocompatibility and robust ROS scavenging activity, GFeSNs exhibited superior therapeutic efficiency towards in vivo ROS-induced AKI. Taken together, these advantageous features suggest that GFeSNs may be promising therapeutic agents for the treatment of AKI and ROS scavengers in biomedical treatment and research.

### Supplementary Information

**Additional file 1: Figure S1.** The release trend of hydrogen polysulfide from GFeSNs. **Figure S2.** Iron ions released from different concentrations of GFeSNs in PBS solution. **Figure S3.** AFM image of GFeSNs and the corresponding height analysis. **Figure S4.** ^•^OH scavenging ratio of the GFeSNs. **Figure S5.** O_2_^•−^ scavenging efficiency and ^•^OH scavenging ratio of GSH. **Figure S6.** O_2_^•−^ scavenging efficiency of GFeSNs after 24 h and 48 h in PBS. **Figure S7.** CAT-like activity of GFeSNs. **Figure S8.** Different enzyme-like activity of GFeSNs under different pH conditions. **Figure S9.** SEM of GFeSNs after dispersed in distilled water for 24 h, 48 h, and 96 h, respectively. **Figure S10.** In vitro hemolysis test of GFeSNs. **Figure S11.** In vivo toxicity evaluation of GFeSNs to major organs (heart, liver, spleen, lung, and kidney) 7 days and 30 days after intravenous administration. **Figure S12.** Serum biochemistry assay and complete blood panel data of mice intravenously injected with PBS or GFeSNs at 24 h.

## Data Availability

All data used to generate these results are available in the main text and supporting information.

## Abbreviations

ROS: Reactive oxygen species
AKI: Acute kidney injury
GSH: Glutathione
NAC: *N*-Acetyl cysteine
AMF: Amifostine
ABTS: 2,2′-Azinobis(3-ethylbenzthiazoline-6-sulfonate)
FDA: Food and Drug Administration
MRI: Magnetic resonance imaging
FeSNs: Iron sulfide nanomaterials
H_2_O_2_: Hydrogen peroxide
DATS: Diallyl trisulfide
·OH: Hydroxyl radical
O_2_^**.**^^−^: Superoxide anion
FeCl_3_: Ferric chloride
TMB: 3,3′,5,5′-Tetramethylbenzidine
DCFH-DA: 2′,7′-Dichlorofluorescein diacetate
EG: Ethylene glycol
DPPH: 1-Diphenyl-2-picrylhydrazyl
DMPO: 5,5-Dimethyl-1-pyrrooline N-oxide
SEM: Scanning electron microscopy
TEM: Transmission electron microscopy
HRTEM: High-resolution transmission electron microscope
STEM-EDX: Scanning transmission electronic microscope-energy dispersive X-ray spectroscopy
SEAD: Selected area electron diffraction
XPS: X-ray photoelectron spectroscopy
ESR: Electron spin resonance
SOD: Superoxide dismutase
CAT: Catalase
SulfoBiotics-SSP4: 3′,6′-Di(O-thiosalicyl)fluorescein
PI: Propidium iodide
JC-1: 5,5′,6,6′-Tetrachloro-1,1′,3,3′-tetraethylimidacarbocyanine
H&E: Hematoxylin and eosin
ALT: Alanine transaminase
ALP: Alkaline phosphatase
AST: Aspartate transaminase
CRE: Creatinine
IL-1β: Interleukin-1β
IL-6: Interleukin-6
TNF-α: Tumor necrosis factor-α
IFN-γ: Interferon-γ
BUN: Blood urea nitrogen
HO-1: Heme oxygenase-1
KIM-1: Kidney injury molecule-1
MDA: Malondialdehyde
LDH: Lactate dehydrogenase
DAPI: 4′,6-Diamidino-2-phenylindole
DHE: Dihydroethidium
TUNEL: Terminal deoxynucleotidyl transferase mediated dUTP nick-end labelling
MB: Methylene blue
PCA: Principal component analysis
DEG: Differentially expressed genes
KEGG: Kyoto Encyclopedia of Genes and Genomes
GSEA: Gene set enrichment analysis
