# Supplementary material for: Mackinawite nanozymes as reactive oxygen species scavengers for acute kidney injury alleviation
Source: J Nanobiotechnology. 2023 Aug 19;21:281. doi: 10.1186/s12951-023-02034-7 (PMC10439570; doi:10.1186/s12951-023-02034-7)
Supplement: Supplementary file 1 — Additional file 1: Figure S1. The release trend of hydrogen polysulfide from GFeSNs. Figure S2. Iron ions released from different concentrations of GFeSNs in PBS solution. Figure S3. AFM image of GFeSNs and the corresponding height analysis. Figure S4. •OH scavenging ratio of the GFeSNs. Figure S5. O2•− scavenging efficiency and •OH scavenging ratio of GSH. Figure S6. O2•− scavenging efficiency of GFeSNs after 24 h and 48 h in PBS. Figure S7. CAT-like activity of GFeSNs. Figure S8. Different enzyme-like activity of GFeSNs under different pH conditions. Figure S9. SEM of GFeSNs after dispersed in distilled water for 24 h, 48 h, and 96 h, respectively. Figure S10. In vitro hemolysis test of GFeSNs. Figure S11. In vivo toxicity evaluation of GFeSNs to major organs (heart, liver, spleen, lung, and kidney) 7 days and 30 days after intravenous administration. Figure S12. Serum biochemistry assay and complete blood panel data of mice intravenously injected with PBS or GFeSNs at 24 h. [file 12951_2023_2034_MOESM1_ESM.docx]

Supporting information for

**Mackinawite** **nanozymes as** **reactive oxygen species scavengers for acute kidney injury alleviation**

Zhuobin Xu^1,2^^†^, Yufei Zhu^1,2†^, Mengke Xie^1^, Kankan Liu^1^, Liangliang Cai^1,2^, Huihui Wang^1,2^, Dandan Li^1,2*^, Hao Chen^1,2,3*^ and Lizeng Gao^4*^

^1^ *Institute of Translational Medicine, Medical College, Yangzhou University, Yangzhou, 225009, China*

^2^ *Jiangsu Key Laboratory of Integrated Traditional Chinese and Western Medicine for Prevention and Treatment of Senile Diseases, Yangzhou University, Yangzhou, 225009, China*

^3^ *Department of Orthopedics, Affiliated Hospital of Yangzhou University, Yangzhou 225009, Jiangsu, China*

^4^ *CAS Engineering Laboratory for Nanozyme, Institute of Biophysics, Chinese Academy of Sciences, Beijing 100101, China*

*Corresponding authors:

Dandan Li (Email: [006544@yzu.edu.cn](mailto:006544@yzu.edu.cn));

Hao Chen (Email: [hchen](mailto:hchen)2020@[yzu.edu.cn](mailto:hchen2020@yzu.edu.cn));

Lizeng Gao (Email: [gaolizeng@ibp.ac.cn](mailto:gaolizeng@ibp.ac.cn))

^†^These authors contributed equally to this work.

*
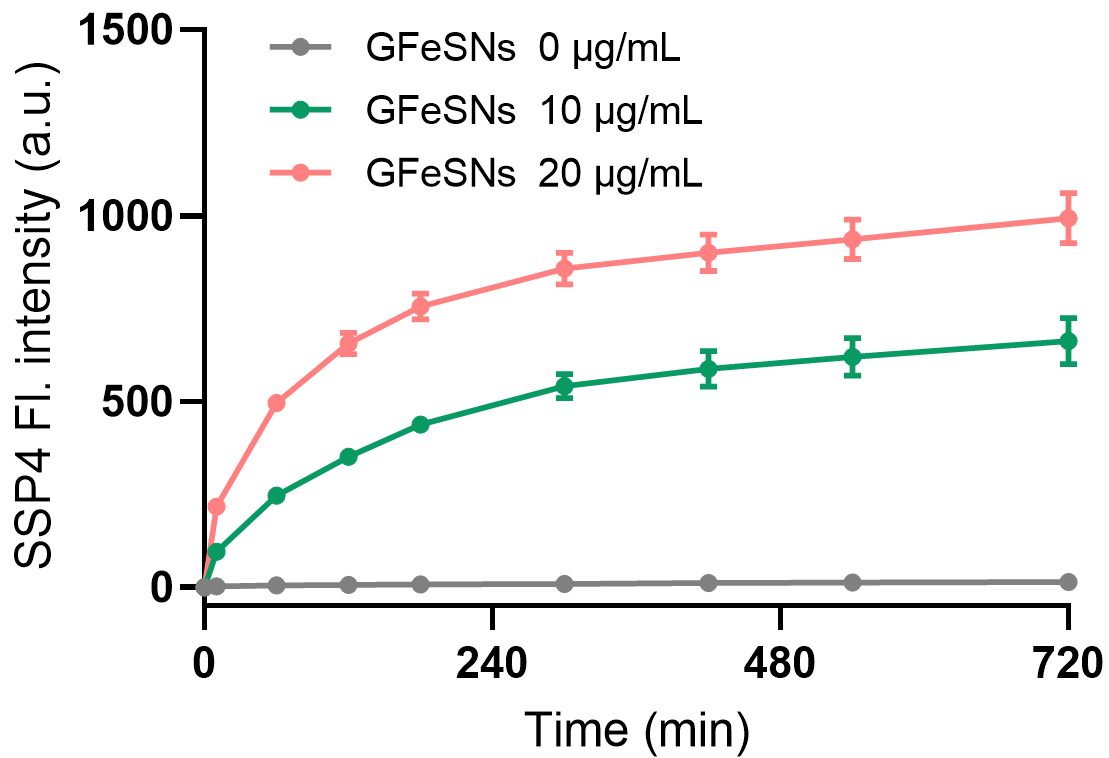
*

**Figure S1.** Release trend of hydrogen polysulfide from GFeSNs. Data represent means ± s.d. from three independent replicates.


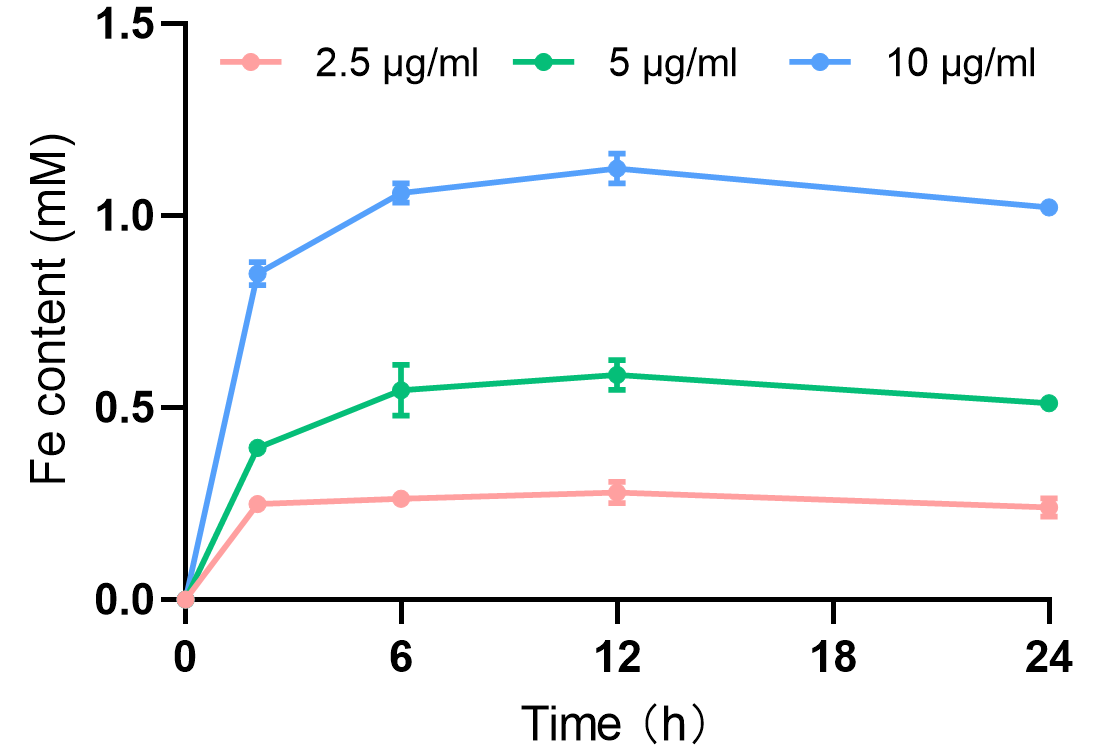


**Figure S2.** Iron ions released from different concentrations of GFeSNs in PBS solution. Data represent means ± s.d. from three independent replicates.


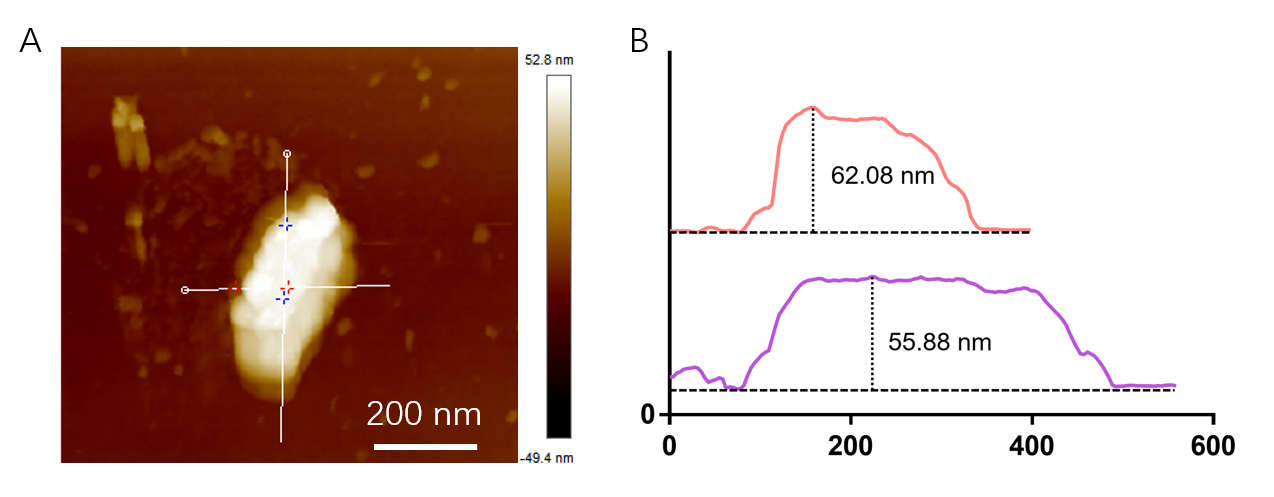


**Figure S3.** AFM image of GFeSNs and the corresponding height analysis.


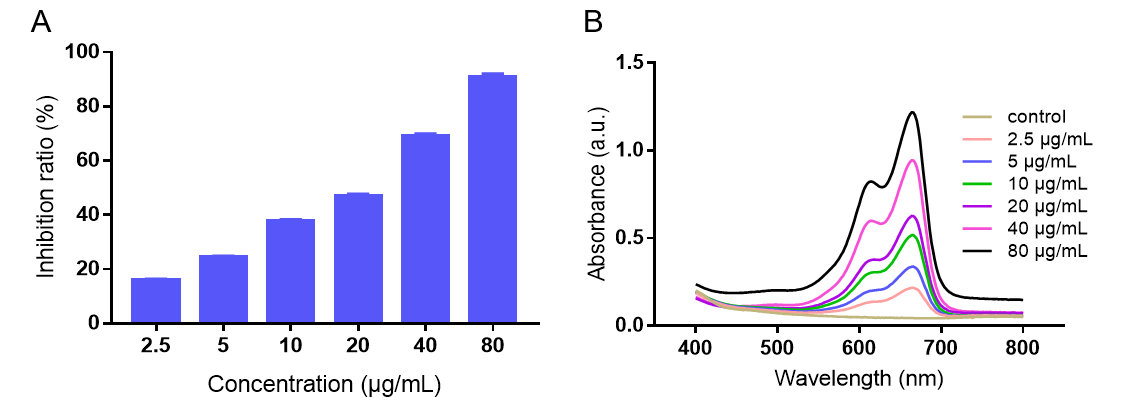


**Figure S4.** ^•^OH scavenging ratio of the GFeSNs. A, Data statistics of ROS scavenging ability based on the UV-vis absorbance of MB. B, The UV-vis absorbance spectra of MB with different treatments. Different concentrations of GFeSNs (0-80 μg/mL) were mixed with MB and added to Fenton reaction solution containing H_2_O_2_ and Fe^2+^. After 15 minutes of reaction, the absorbance of the solution was measured, and the ^•^OH scavenging abilities were calculated.

**
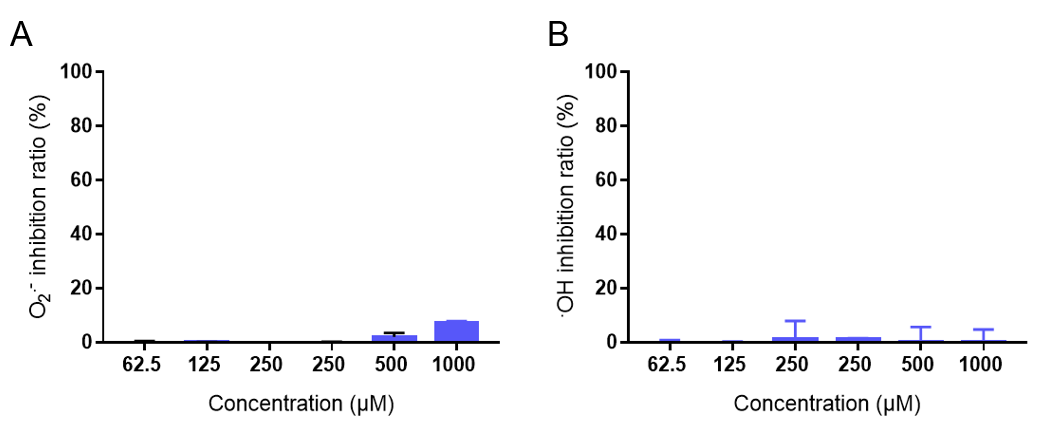
**

**Figure S5.** A and B, O_2_^•-^ scavenging efficiency and ^•^OH scavenging ratio of GSH. The concentration for GSH was normalized to sulfur amount equal to that contained in the GFeSNs from 2.5-80 μg/mL.


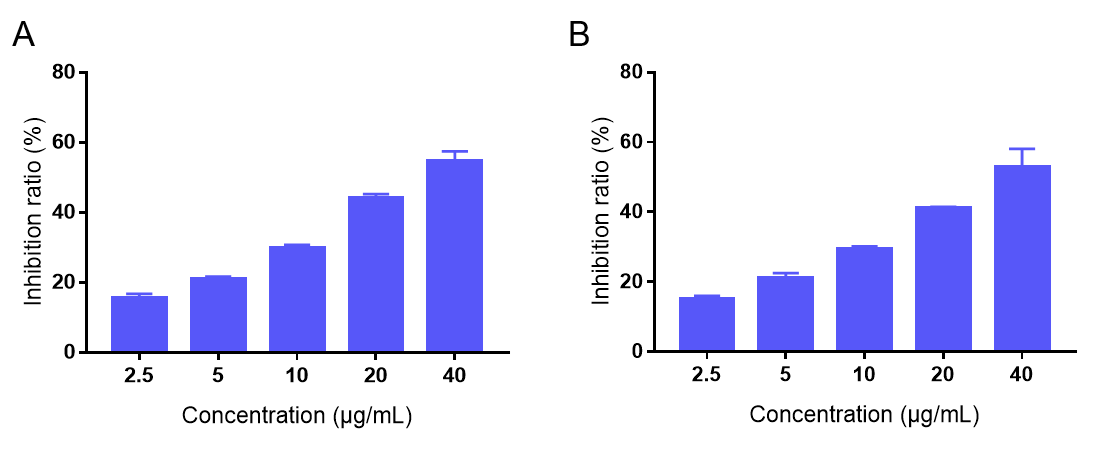


**Figure S6.** O_2_^•-^ scavenging efficiency of GFeSNs after 24 h (A) and 48 h (B) in PBS. GFeSNs were dissolved in PBS, and O_2_^•-^ scavenging ability of GFeSNs was measured by SOD kit at 24 h and 48 h, respectively.


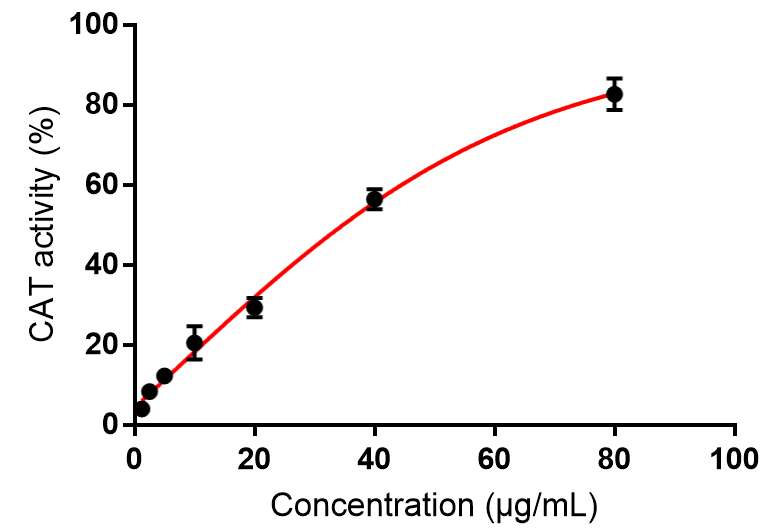


**Figure S7.** CAT-like activity of GFeSNs. Catalase catalyzes the production of water and oxygen from hydrogen peroxide. The residual hydrogen peroxide can oxidize the chromogenic substrate, resulting in the red product N-(4-antipyryl)-3-chloro-5-sulfonate-p-benzoquinone monoamine under the catalysis of peroxidase, and then the maximum absorption wavelength of 520 nm was recorded. Specifically, different concentrations of GFeSNs (0-80 μg/mL) were incubated with 250 mM H_2_O_2_ at 25 ℃ for 5 minutes, and then chromogenic agent was added to detect the absorbance at 520 nm.


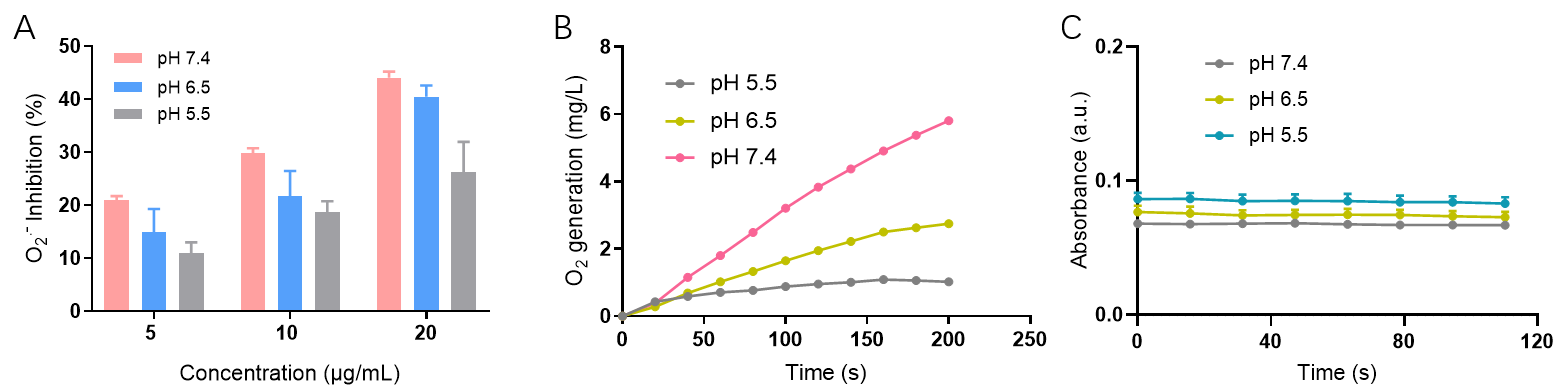


**Figure S8.** Different enzyme-like activity of GFeSNs under different pH conditions. (A) O_2_^•-^ scavenging efficiency of GFeSNs at pH 7.4/6.5/5.5. (B) O_2_ Generation by GFeSNs (10 μg/mL) in 800 mM H_2_O_2_ solution at pH 7.4/6.5/5.5. (C) Effects of GFeSNs on peroxide elimination: GFeSNs (10 μg/mL), H_2_O_2_ (20 mM), TMB (0.4 mM).


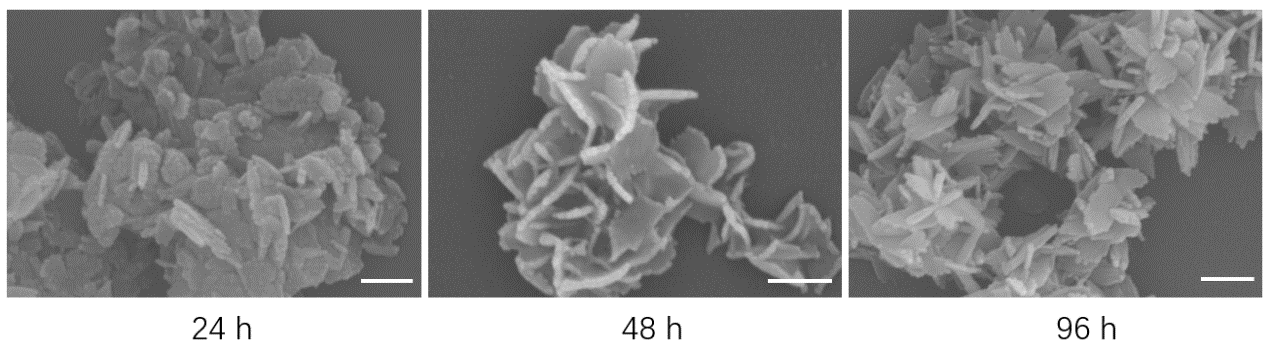


**Figure S9.** SEM of GFeSNs after dispersed in distilled water for 24 h, 48 h, and 96 h, respectively. Scale bar: 200 nm.


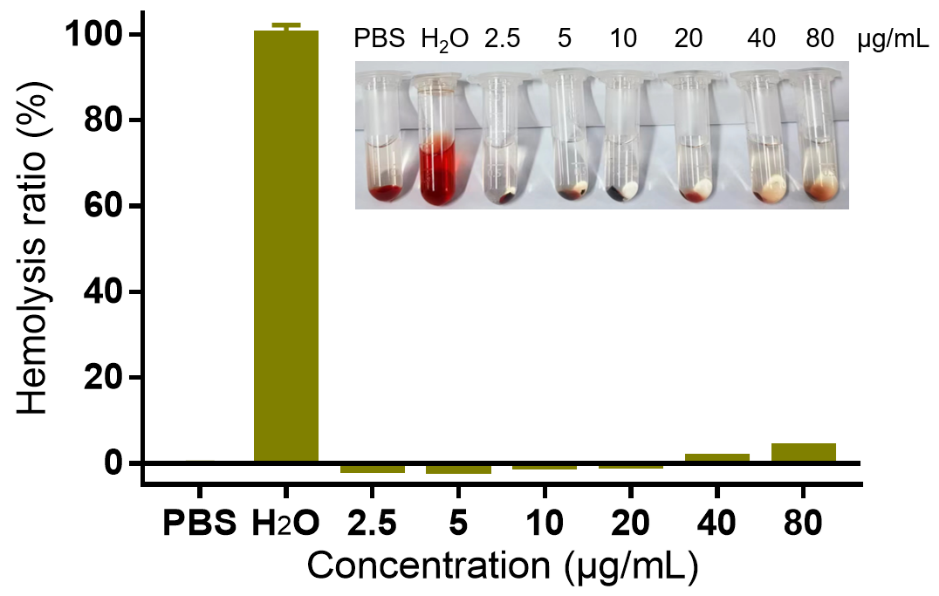


**Figure S10.** In vitro hemolysis test of GFeSNs. Insets are digital photographs of hemolysis for GFeSNs with different concentrations. In brief, whole blood was centrifuged at 1000 rpm for 10 min, red blood cells were collected and gently washed three times with PBS solution. Then, the diluted red cell suspension was mixed with GFeSNs (0-80 μg/mL). The mixed dispersions were incubated at 37°C for 3 h, centrifuged at 3500 rpm for 5 min, and hemolysis was observed and recorded.


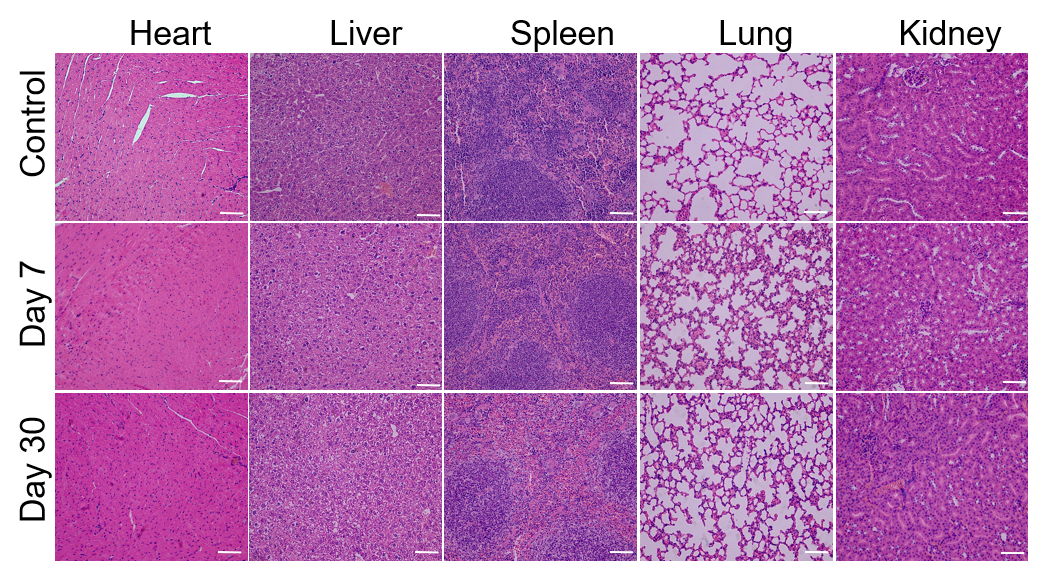


**Figure S11.** In vivo toxicity evaluation of GFeSNs to major organs (heart, liver, spleen, lung, and kidney) 7 days and 30 days after intravenous administration. Scale bar: 50 μm.

**
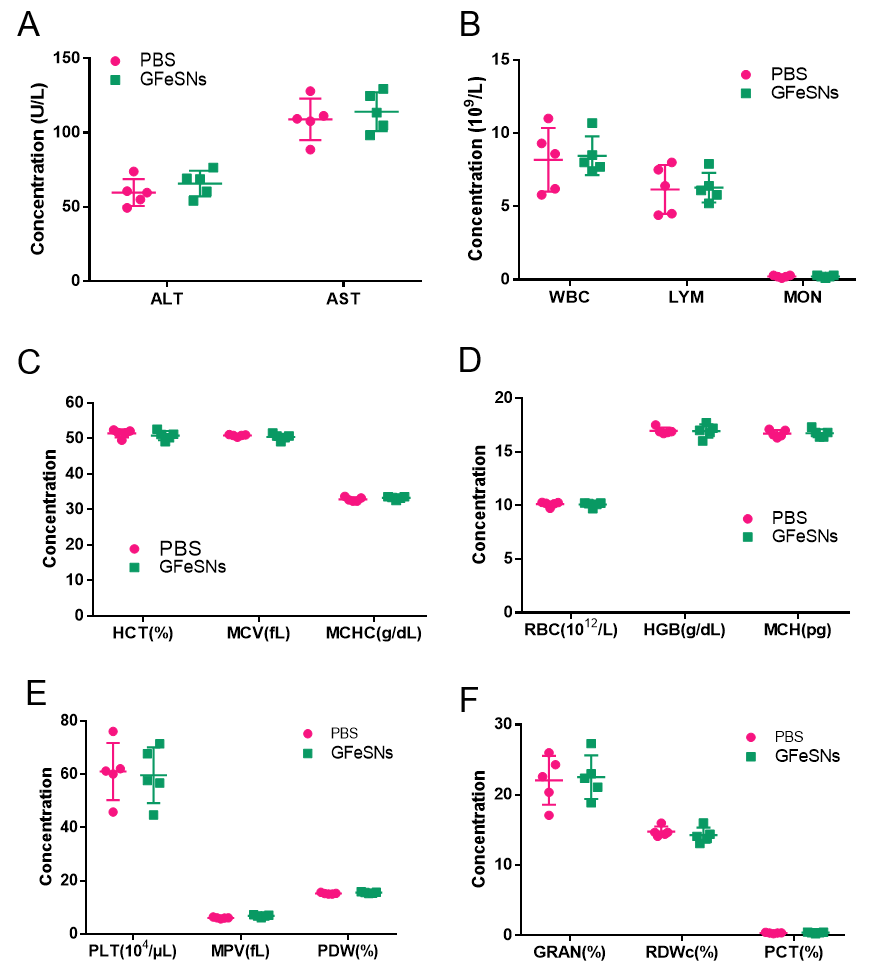
**

**Figure S12.** (a-f) Serum biochemistry assay and complete blood panel data of mice intravenously injected with PBS or GFeSNs at 24 h. Data represent means ± s.d. from five independent replicates.
